# A comprehensive analysis of penile cancer in the region with the highest worldwide incidence reveals new insights into the disease

**DOI:** 10.1186/s12885-022-10127-z

**Published:** 2022-10-15

**Authors:** Antonio Augusto Lima Teixeira Júnior, Syomara Pereira da Costa Melo, Jaqueline Diniz Pinho, Thaís Bastos Moraes Sobrinho, Thalita Moura Silva Rocha, Denner Rodrigo Diniz Duarte, Liseana de Oliveira Barbosa, Wesliany Everton Duarte, Marta Regina de Castro Belfort, Kelly Gomes Duarte, Antonio Lima da Silva Neto, José de Ribamar Rodrigues Calixto, Lúcio Cristiano Paiva Paiva, Francisco Sérgio Moura Silva do Nascimento, Antonio Machado Alencar Junior, André Salim Khayat, Rita da Graça Carvalhal Frazão Corrêa, Joyce Santos Lages, Rodolfo Borges dos Reis, Wilson Silva Araújo, Gyl Eanes Barros Silva

**Affiliations:** 1 Department of Genetics and Postgraduate Program in Genetics, Ribeirão Preto Medical School, University of São Paulo, Ribeirão Preto, Brazil; 2 Postgraduate Program in Adult Health, Federal University of Maranhão, São Luís, Brazil; 3 Laboratory of Immunofluorescence and Electron Microscopy, University Hospital of the Federal University of Maranhão, São Luís, Brazil; 4 Higher Education Center of Zé Doca, State University of Maranhão, Zé Doca, Brazil; 5 Postgraduate Program in Health Science, Federal University of Maranhão, São Luís, Brazil; 6 Postgraduate Program in Clinical Surgery, Ribeirão Preto Medical School, University of São Paulo, Ribeirão Preto, Brazil; 7 University Hospital of the Federal University of Maranhão, São Luís, Brazil; 8 Oncology Research Center, Federal University of Pará, Belém, Brazil

**Keywords:** Penile cancer, HPV, p16, p53, Ki-67, Maranhão, Brazil

## Abstract

**Background:**

Although penile cancer (PC) is uncommon in developed countries, it is widespread in developing countries. The state of Maranhão (Northeast, Brazil) has the highest global incidence recorded for PC, and, despite its socioeconomic vulnerability, it has been attributed to human papillomavirus (HPV) infection. This study aimed to determine the histopathological features, the prevalence of HPV infection, and the immunohistochemical profile of PC in Maranhão.

**Methods:**

A retrospective cohort of 200 PC cases were evaluated. HPV detection was performed using nested-PCR followed by direct sequencing for genotyping. Immunohistochemistry (IHC) was performed using monoclonal antibodies anti-p16^INK4a^, p53, and ki-67.

**Results:**

Our data revealed a delay of 17 months in diagnosis, a high rate of penile amputation (96.5%), and HPV infection (80.5%) in patients from Maranhão (Molecular detection). We demonstrated the high rate of HPV in PC also by histopathological and IHC analysis. Most patients presented koilocytosis (75.5%), which was associated with those reporting more than 10 different sexual partners during their lifetime (*p* = 0.001). IHC revealed frequent p16^INK4a^ overexpression (26.0%) associated with basaloid (*p* < 0.001) and high-grade tumors (*p* = 0.008). Interestingly, p16 appears not to be a better prognostic factor in our disease-free survival analysis, as previously reported. We also demonstrated high ki-67 and p53 expression in a subset of cases, which was related to worse prognostic factors such as high-grade tumors, angiolymphatic and perineural invasion, and lymph node metastasis. We found a significant impact of high ki-67 (*p* = 0.002, log-rank) and p53 (*p* = 0.032, log-rank) expression on decreasing patients’ survival, as well as grade, pT, stage, pattern, and depth of invasion (*p* < 0.05, log-rank).

**Conclusions:**

Our data reaffirmed the high incidence of HPV infection in PC cases from Maranhão and offer new insights into potential factors that may contribute to the high PC incidence in the region. We highlighted the possible association of HPV with worse clinical prognosis factors, differently from what was observed in other regions. Furthermore, our IHC analysis reinforces p16, ki-67, and p53 expression as important diagnosis and/or prognosis biomarkers, potentially used in the clinical setting in emerging countries such as Brazil.

**Supplementary Information:**

The online version contains supplementary material available at 10.1186/s12885-022-10127-z.

## Background

Penile cancer (PC) is an uncommon neoplasm in developed countries. However, it has a high incidence in developing countries, mainly in Asia, Africa, and South America [1, 2]. When compared to those of other cancers, the epidemiological, etiological, and histopathological aspects of PC are poorly characterized.

Nevertheless, some risk factors have been proposed for PCs. The disease appears to be more prevalent in older men living in rural zones and those with low education levels [3]. PC has been linked to smoking, alcoholism, phimosis, obesity, poor hygiene of the penis, chronic inflammatory conditions, and human papillomavirus (HPV) infection [4].

According to data from the International Agency for Research on Cancer (IARC), the age-standardized rate (ASR) of PC in the United States is 0.5/100.000 men [5]. It is even lower for Jewish men born in Israel (0.1/100.000 men). The low age-standardized rate reported in Israel is associated with the cultural practice of neonatal circumcision, which appears to be a protective factor against the disease.

In contrast, the highest incidence was reported in Uganda, Africa (5.6 per 100,000 men). According to the IARC, Brazil is ranked thirteenth worldwide for incidence of PC, with a rate of 1.6/100.000 men [5]. However, the IARC only took six cities from Brazil into account to calculate the national incidence: Aracaju, Belo Horizonte, Cuiabá, Fortaleza, Goiânia, and São Paulo. Most of these cities (four out of six) are not in the poorest areas of Brazil (where PC is more common), so these data may not accurately portray the incidence of the disease in Brazil.

Since the prevalence of PC appears to vary considerably between certain regions of Brazil, local studies are essential to better understand the reality of PC in the country. These studies reveal the North and Northeast of Brazil as the regions with the highest incidence of the disease, where according to Korkes et al. 2020, between 2006 and 2018 the incidence of PC in these regions was 5.4 per 100,000 men [6].

In northeastern Brazil, the state of Maranhão has attracted the attention of the scientific community owing to its high prevalence of PC [7]. In 2018, our research group published a retrospective study of 392 patients diagnosed with PC between 2004 and 2014 in Maranhão [8] exceeds the highest national and international rates reported by IARC, revealing an alarming situation of PC in Maranhão.

Our research group and others have aimed to better understand PC features in Maranhão. Over the past five years, these studies have drawn attention to a clinical, epidemiological, and etiological profile distinct from that observed in other studies carried out in Brazil and worldwide, with marked heterogeneity of histological subtypes and high prevalence of HPV infection [7, 9–13].

Therefore, the present study proposes a panoramic analysis of PC in Maranhão, describing the clinical, histopathological, HPV status, and immunohistochemical (IHC) features for diagnostic and/or prognostic importance markers.

## Methods

### Study design

This was a cross-sectional, experimental, and retrospective study. A survey of cases diagnosed with PC between 2006 and 2020 was carried out in two reference hospitals (University Hospital of the Federal University of Maranhão (HU-UFMA) and Hospital do Câncer Aldenora Bello (HCAB)), both located in São Luís, Maranhão, Brazil. Only patients 18 years of age or older with clinical and histopathological PC diagnosis who underwent surgery, and who had tissues available for histological review were included in the study. Patients with clinical and histopathological diagnoses of PC performed under other services and with materials in inadequate conditions for analysis were excluded.

A total of 236 cases of PC (2006–2020) were identified; however, only 200 cases met the inclusion criteria. The study involved a review of clinical and histopathological findings, HPV detection and genotyping, and protein expression profiling by IHC. All clinical data described were collected from medical records. Histopathological and IHC analyzes were performed at the Laboratory of Immunofluorescence and Electronic Microscopy (LIME/HU-UFMA), and molecular assays were performed at the Clinical Research Center (CEPEC) of the HU-UFMA, São Luís, Maranhão, Brazil, and in the Nucleus of Research in Oncology (NPO), at the Federal University of Pará (UFPA), Belém, Pará, Brazil.

Both for histopathological and molecular analysis we used formalin-fixed paraffin-embedded (FFPE) tumor tissue, which was stored under appropriate conditions in the archives of the pathology service of each hospital included in the study.

### Histopathological review

Specimens (*n* = 200) were subjected to hematoxylin and eosin (H&E) staining to confirm the histological diagnosis and classification of tumors according to the criteria of the classification of malignant tumors (TNM) in the American Joint Committee on Cancer (AJCC), 8th edition. All tumors were independently evaluated by three pathologists (SPCM, LOB, and GEBS). In cases of disagreement, the criteria were reviewed a second time.

### Morphological changes suggestive of HPV infection

Histological diagnosis of HPV was performed by identifying the koilocytes. For koilocyte analysis, the three main criteria as previously described [14] were considered: (1) presence of perinuclear halo; (2) nuclear atypia, and (3) presence of multinucleation.

### Tumor DNA extraction

For tumor DNA extraction from formalin-fixed paraffin-embedded (FFPE) tissue, three 10 µm cuts of each paraffin-embedded tissue were made in a histological microtome and transferred to a 2.0 ml microtube. Then, 1.0 µL of xylol was added to the tube, vortexed, and incubated in a thermoblock for 5 min at 50 °C to remove excess paraffin. The samples were centrifuged at 14,000 rpm for 2 min, and the supernatant was discarded. The xylol-washing process was repeated twice. Then 1.0 mL of absolute ethanol was added to each sample, centrifuged again at 14,000 rpm for 2 min, the supernatant was discarded, and the ethanol wash was repeated two more times. Samples were then incubated at 37ºC for 40 min to dry all alcohol in the sample. DNA isolation was done with the QIAamp DNA FFPE Tissue Kit (Qiagen, Hilden, Germany), according to the manufacturer’s instructions. Each sample was diluted in 50 µL of RNase-free TE, pH 8.0, and quantified using a NanoDropOne® spectrophotometer (Thermo Fisher Scientific, Waltham, USA), with concentrations expressed in ng/µL, and purity analysis with measures 260/280 (between 1.8 and 2.0) and 260/230 (above one). DNA samples were immediately used for molecular detection of HPV or stored at -20 °C.

### HPV molecular detection

DNA extraction resulted in only 113 samples within the expected quality standards, so HPV detection was performed only in these cases. Each sample was subjected to PCR (Polymerase Chain Reaction) for the β-globin endogenous gene as second quality control. All samples were β-globin positive. HPV detection was performed by nested-PCR. In the first PCR, a set of generic primers called PGMY09/11 was used, which produced a 450 bp amplicon of the L1-HPV capsid region [15]. In the second PCR, GP5 + /6 + primers were used; which generate a 170 bp amplicon contained in the 450 bp fragment amplified in the first PCR [16]. All primer sequences and conditions for each PCR are described in the tables available in the supplementary material (S1). Amplicons were separated on a 1.5% agarose gel and visualized. Those with amplification for both PGMY09/11 and GP5 + /6 + were considered HPV-positive.

For HPV genotyping, the second PCR amplicons of HPV-positive cases were sequenced in a single direction using the GP5 + primer. Automatic sequencing was performed on the ABI PRISM 3500XL Genetic Analyzer, using the BigDye™ Terminator v3.1 Cycle Sequencing kit (Applied Biosystems™). The sequences obtained were uploaded to the MEGA 6.0 and compared with those from the GeneBank/NCBI database using the Basic Local Alignment Search Tool (BLAST) for genotyping (http://blast.ncbi.nlm.nih.gov/Blast.cgi).

### Immunohistochemistry

IHC analysis was performed on 3 µm-thick sections from FFPE tumor tissue from 173 patients, using the EnVision FLEX Mini Kit High pH System (Agilent Technologies, Santa Clara, CA, USA). Antigen retrieval was performed using PT Link equipment (Agilent Technologies) following the manufacturer's instructions. Slides were next washed in Dako Wash Buffer, blocking of endogenous peroxidase was done for 5 min, primary monoclonal antibody for each evaluated marker was applied for 60 min, polymer application (20 min), and staining in chromogen 3,3’ diaminobenzidine (DAB) for 10 min, counterstaining with Harris hematoxylin (5 min), and analysis under an optical microscope. Table 1 describes the specifications of the monoclonal antibodies used in this study. For IHC, first, we performed immunostaining for ki-67, and 173 samples had positive internal control (lymphocytes). Cases that were negative for ki-67 internal control (*n* = 27) were excluded.

**Table 1:** Monoclonal antibodies used in immunohistochemical assays

*Antibody*	*Clone*	*Specie*	*Provider*	*Dilution*	*Staining*
Anti-p16 ^INK4a^	JC2	Mouse	Cell Marque	1: 200^a^	Nuclear/cytoplasmic
Anti-p53	DO-7	Mouse	Dako	Ready to use	Nuclear
Anti-ki67	MIB-1	Mouse	Dako	Ready to use	Nuclear

^a^ Dilutions were performed using EnVision™ FLEX Antibody Diluent, provided by Agilent—Dako (Santa Clara, Ca, USA), Cat. N° K800621-2

Immunostaining was measured semi-quantitatively by two pathologists independently (GEBS and SPCM), and when in disagreement, the slides were reviewed together for consensus. For p16^INK4a^, the Cubilla et al. (2011) criteria were used, which proposed the following categories: (0) complete absence of p16^INK4a^ staining in all tumor cells; (1) spotty, patchy, and discontinuous staining in some of the tumor cells; (2) more extensive but discontinuous staining pattern with small clusters of positive tumor cells; (3) cytoplasmic or nuclear staining in all tumor cells, except in hyperkeratotic or parakeratotic areas when present [17]. Only cases with pattern 3 were considered positive for p16^INK4a^.

For p53, cases with < 20% of tumor cells stained were considered negative, 20-50% were classified as positive ( +), and > 50% of tumor cells were positive (+ +) [18]. For ki-67, cases were classified as low (< 40.5%) and high (> 40.5%) expression, being performed globally (analyzing the entire tumoral slide extent) and in hotspots of proliferating cells [19].

### Disease-free survival

For DFS analysis, only cases with clear information in the medical record (beginning and end of follow-up, and clinical outcome) were included. Only 134 cases met the inclusion criteria. We defined the time of diagnosis as the beginning of follow-up and noted the events of recurrence and/or lymph node metastasis during the analysis period. We considered recurrence when the tumor returned after the first surgical treatment and a period during which it could not be detected.

### Statistical analysis

Data were tabulated using Excel (Microsoft Office 2019) and statistically analyzed using the SPSS® software v. 22 (IBM Corp., Armonk, NY, USA). The association analysis between clinical, histopathological, immunohistochemical, and HPV infection variables was performed using bivariate tests (chi-square test or Fisher's exact test). The Kaplan–Meier method was used to estimate the DFS, and the log-rank test was applied to compare the survival curves. For all analyses, results with *p* < 0.05 were considered significant.

## Results

### Social, demographic, and clinical data

**Fig. 1:**
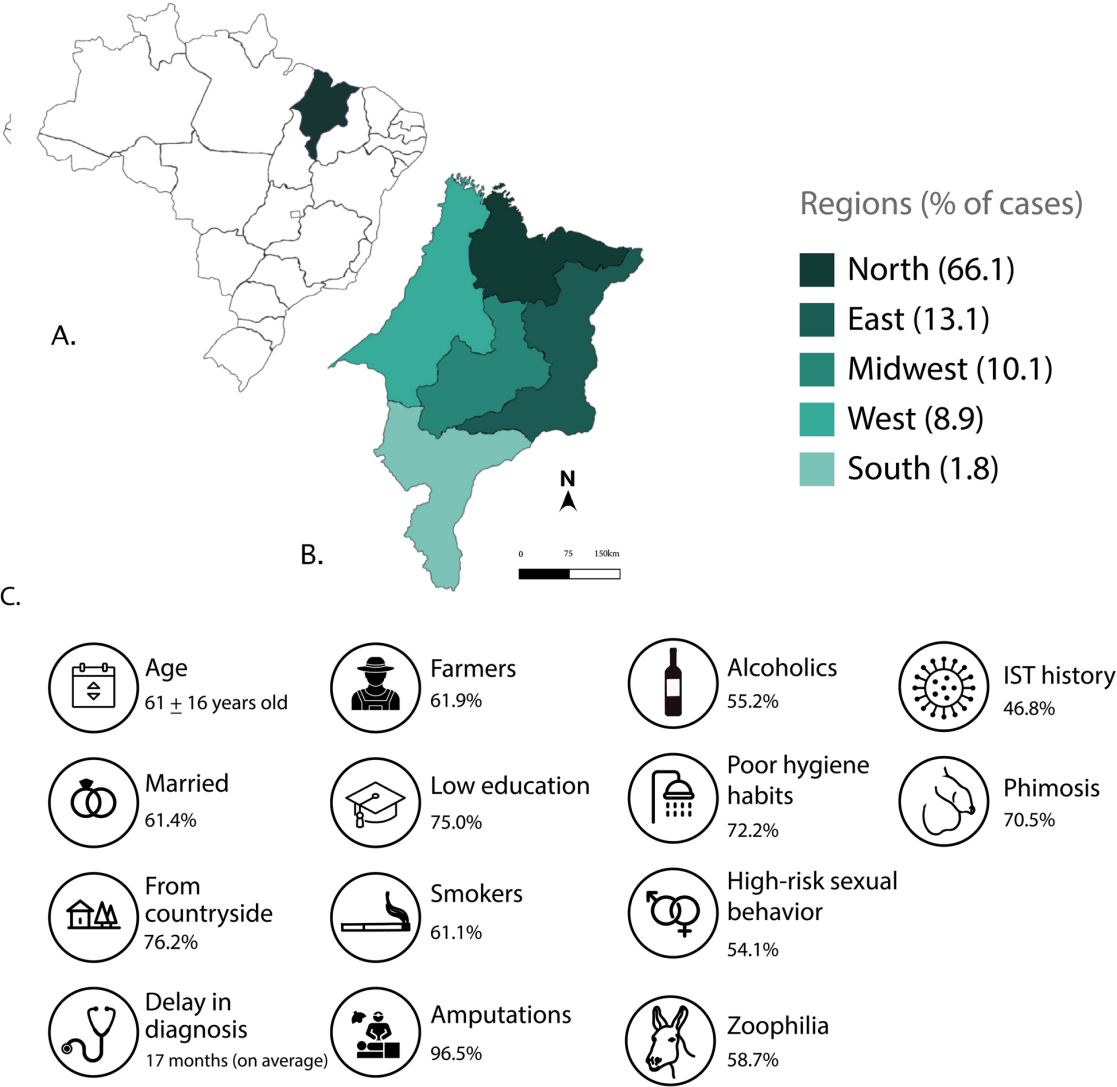
Panoramic view of PC in Maranhão, Northeast Brazil. (**A**) Map of Brazil highlighting the state of Maranhão in green; (**B**) Distribution of PC cases in Maranhão according to the five main regions of the state; (**C**) Primary socio-behavioral and clinical characteristics of the 200 PC cases from Maranhão analyzed in this study

In this study, we evaluated 200 patients with clinical and pathological diagnoses of PC treated in the state of Maranhão, Brazil, between 2006 and 2020. Of these, 40 (20.0%) were admitted to the HU-UFMA and 160 (80.0%) were admitted to the HCAB. Figure 1 describes the primary socio-behavioral and clinical characteristics of these patients and their geographical distribution in Maranhão. Their ages ranged from 23 to 95 years (mean 61 + 16 years old), and there was a prevalence of men with low education (75.0% never attended school or had incomplete primary education).

There was a high frequency of patients with risky sexual behavior, with 54.1% reporting more than 10 different sexual partners during their lifetime (mostly having sex without condoms), and 58.7% practicing zoophilia (sex with animals). Among those who reported a history of sexually transmitted infections (STIs), gonorrhea was the most frequent (67.6%). Only one patient tested positive for the human immunodeficiency virus (HIV). Among those who reported phimosis, 43.0% underwent circumcision in adulthood, usually preceded by surgical treatment of the tumor.

### Surgery and histopathology

Surgical treatment was performed in all cases, mainly partial penis amputation (72.5%). The main affected topography was the glans (91.9%), either individually (44.4%), or simultaneously with the foreskin and/or coronal sulcus (27.3%) or penile body (20.2%). The tumor size ranged from 0.6 cm to > 10.0 cm, the majority being between 2.0 and 5.0 cm (61.9%). Tumors were unifocal in 89.9% of cases. An ulcerated aspect occurred in 30.8%, or ulcer-vegetating in 25.8%, and with an expansive pattern of growth in 75.3%.

In H&E analysis, all tumors were squamous cell carcinomas (SCC), poorly differentiated in 49.0% of cases, and mainly of the usual (39.5%) and warty (29.0%) subtypes. The presence of carcinoma in situ associated with invasive lesion was observed in 82.0% of cases, and lichen sclerosus in 27.3%. Angiolymphatic and perineural involvement was present in 35.5% and 36.5% of cases, respectively, and sarcomatoid dedifferentiation in 17.1% of cases.

We also evaluated the thickness and depth of tumor invasion. We considered “thickness” as the measure between the largest point on the tumor surface and the deepest point of the infiltration front and as “depth” the distance between the basement membrane and the deepest point of infiltration. Slight majorities had a thickness > 5.0 cm (61.0%) and depth < 5.0 cm (52.4%). According to the TNM classification, these tumors were mainly in stage pT3 (48.0%), pNx (92.5%), and pMx (100.0%). Pathological stage IIb at diagnosis was seen in 44.0% of cases. The complete characterization of the histopathological parameters is presented in Table 2.

**Table 2:** Histopathological aspects evaluated according to each subgroup

Parameters	Total *n* = 200	HPV *n* = 113	IHC *n* = 173
***Surgical procedure***	**N (%)**	**N (%)**	**N (%)**
Preservative (exeresis)	7 (3.5)	0 (0.0)	5 (2.9)
Total glansectomy	7 (3.5)	4 (3.5)	7 (4.0)
Partial penectomy	145 (72.5)	80 (70.8)	126 (72.8)
Total penectomy	28 (14.0)	19 (16.8)	24 (13.9)
Emasculation	13 (6.5)	10 (8.8)	11 (6.4)
***Topography***			
Foreskin and/or coronal sulcus	8 (4.0)	2 (1.8)	7 (4.1)
Glans	88 (44.4)	41 (36.3)	75 (43.6)
Corpus	7 (3.5)	3 (2.7)	5 (2.9)
Glans + foreskin and/or coronal sulcus	54 (27.3)	38 (33.6)	47 (27.3)
Glans + corpus	40 (20.2)	29 (25.6)	37 (21.5)
Others	1 (0.5)	0 (0.0)	1 (0.6)
No information	2	0	1
***Size (cm)***			
0.6 – 2.0	18 (9.3)	9 (8.0)	17 (10.1)
2.1 – 5.0	120 (61.9)	64 (57.1)	100 (59.2)
5.1 – 10.0	54 (27.8)	37 (33.0)	50 (29.6)
> 10.0	2 (1.0)	2 (1.8)	2 (1.2)
No information	6	1	4
***Macroscopic aspects***			
Ulcerative	61 (30.8)	34 (30.1)	50 (29.1)
Vegetative	46 (23.2)	34 (30.1)	44 (25.6)
Verrucous	11 (5.6)	7 (6.2)	9 (5.2)
Ulcerative-vegetative	51 (25.8)	28 (24.8)	44 (25.6)
Others	29 (14.6)	10 (8.8)	25 (14.5)
No information	2	0	1
***Histological type***			
Squamous cell carcinoma	200 (100.0)	113 (100.0)	173 (100.0)
***Histological subtype***			
Usual	79 (39.5)	44 (38.9)	65 (37.6)
Warty	58 (29.0)	32 (28.3)	52 (30.1)
Basaloid	8 (4.0)	6 (5.3)	8 (4.6)
Warty-basaloid	17 (8.5)	8 (7.1)	14 (8.1)
Mixed^a^	32 (16.0)	21 (18.6)	30 (17.3)
Others^b^	6 (3.0)	2 (1.8)	4 (2.3)
***Grade (G)***			
G1	25 (12.5)	14 (12.4)	20 (11.6)
G2	77 (38.5)	36 (31.9)	65 (37.6)
G3	98 (49.0)	63 (55.8)	88 (50.9)
***Angiolymphatic invasion***			
Detected	71 (35.5)	47 (41.6)	64 (37.0)
Not detected	129 (64.5)	66 (58.4)	109 (63.0)
***Perineural invasion***			
Detected	73 (36.5)	45 (39.8)	64 (37.0)
Not detected	127 (63.5)	68 (60.2)	109 (63.0)
***Tumor focus***			
Unifocal	178 (89.9)	97 (85.8)	152 (88.4)
Multifocal	20 (10.1)	16 (14.2)	20 (11.6)
No information	2	0	1
***Carcinoma *in situ*—associated***			
Present	164 (82.0)	92 (81.4)	143 (82.7)
Absent	36 (18.0)	21 (18.6)	30 (17.3)
***Sarcomatoid component***			
Present	34 (17.1)	24 (21.2)	31 (17.9)
Absent	165 (82.9)	89 (78.8)	142 (82.1)
No information	1	0	0
***Lichen sclerosus***			
Present	53 (27.3)	27 (24.5)	44 (26.0)
Absent	141 (72.7)	83 (75.5)	125 (74.0)
No information	6	3	4
***Koilocytosis***			
Present	157 (78.5)	97 (85.8)	140 (80.9)
Absent	43 (21.5)	16 (14.2)	33 (19.1)
***Primary tumor (T)***			
pT1a	42 (21.0)	16 (14.2)	35 (20.2)
pT1b	3 (1.5)	1 (0.9)	3 (1.7)
pT2	53 (26.5)	31 (27.4)	46 (26.6)
pT3	96 (48.0)	61 (54.0)	84 (48.6)
pT4	6 (3.0)	4 (3.5)	5 (2.9)
***Stage***			
I	42 (21.0)	16 (14.2)	35 (20.2)
IIa	53 (26.5)	30 (26.5)	46 (26.6)
IIb	88 (44.0)	55 (48.7)	77 (44.5)
IIIa	1 (0.5)	0 (0.0)	1 (0.6)
IIIb	1 (0.5)	1 (0.9)	1 (0.6)
IV	15 (7.5)	11 (9.7)	13 (7.5)
***Pattern of invasion***			
Expansive	140 (75.3)	79 (73.8)	124 (75.6)
Infiltrative	44 (23.7)	26 (24.3)	38 (23.2)
Others	2 (1.1)	2 (1.9)	2 (1.2)
No information	14	6	9
***Tumor thickness (mm)***			
≤ 5.0	73 (39.0)	39 (35.8)	68 (41.2)
> 5.0	114 (61.0)	70 (64.2)	97 (58.8)
No information	13	4	8
***Depth of invasion (mm)***			
≤ 5.0	97 (52.4)	53 (49.1)	89 (54.3)
> 5.0—≤ 10.0	61 (33.0)	35 (32.4)	51 (31.1)
> 10.0	27 (14.6)	20 (18.5)	24 (14.6)
No information	15	5	9

^a^ Mixed tumors were: 84.4% usual and warty (*n* = 27); 6.3% usual, warty, and basaloid (*n* = 2); 6.3% usual and warty-basaloid (*n* = 2); 3.1% usual and basaloid (*n* = 1)

^b^ Other tumors were: 66.6% were pseudohiperplastic (*n* = 4); 16.7% medullary (*n* = 1); 16.7% papilar (*n* = 1)

The main complaints reported were the appearance of a nodule/tumor in the penis (46.5%); ulcerated wound (21.0%), secretion (12.3%), nodule with presence of secretion (7.9%), dysuria and/or oliguria (7.0%), and other symptoms, such as a burning sensation, discomfort in the penile region, or itching (5.3%). These patients took an average of 17 months to seek medical help from the onset of symptoms (ranging from 1 to 96 months; two cases were excluded as they reported a delay of 180 and 240 months) and usually with locally advanced tumors (pT3-T4, 51.0%).

**Fig. 2:**
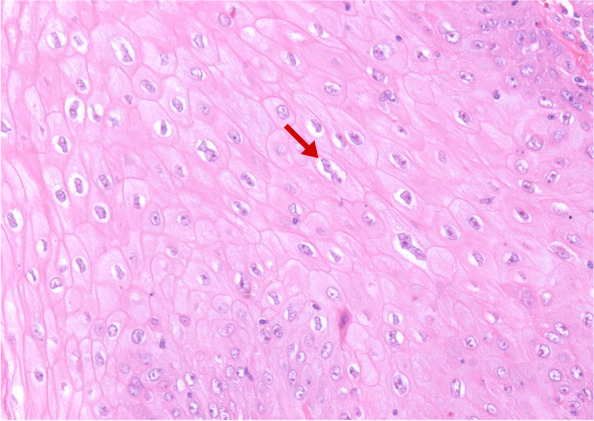
Histological section of penile cancer in HE representing koilocytes (magnification 100x)

Additionally, we identified histological alterations suggestive of HPV infection (koilocytosis) in 78.5% of cases (Fig. 2). The presence of koilocytosis was associated with individuals who reported more than 10 lifetime sexual partners (*p* = 0.001), warty tumors (*p* < 0.0001), HPV-associated histological subtypes (*p* < 0.0001), and p53 negativity (*p* = 0.007).

Physical examination of palpable lymph nodes was performed in 102 of the 200 cases evaluated (obtained from medical records). Of these, 61 (59.8%) had palpable lymph nodes unilaterally or bilaterally, 47 (46.1%) at admission, and 14 (13.7%) after tumor amputation. 74 cases underwent inguinal lymphadenectomy, which was performed unilaterally in 32 cases (43.2%) and bilaterally in 42 cases (56.8%). The presence of lymph node metastasis was confirmed in the histopathological analysis in 47 cases (63.5%), and 13 (27.6%) were simultaneous with primary tumor amputation/diagnosis. Extracapsular involvement was observed in 37 (78.7%) of the 74 patients with lymph node metastasis. The lymph node involvement data are presented in Table 3.

**Table 3:** Clinical and histopathological features of lymph node metastasis in penile cancer patients

Parameters	N (%)
*Palpable lymph nodes (n* = *102)*	
Yes	61 (59.8)
No	41 (40.2)
No information	98
*Type of surgery (n* = *74)*	
Unilateral	32 (43.2)
Bilateral	42 (56.8)
No lymphadenectomy	126
*Lymph node metastasis (n* = *74)*	
Present	47 (63.5)
Absent	27 (36.5)
No lymphadenectomy	126
*Extradonal extension (n* = *47)*	
Present	37 (77.1)
Absent	11 (22.9)
No metastasis or no lymphadenectomy	152

### HPV Molecular Detection

**Fig. 3:**
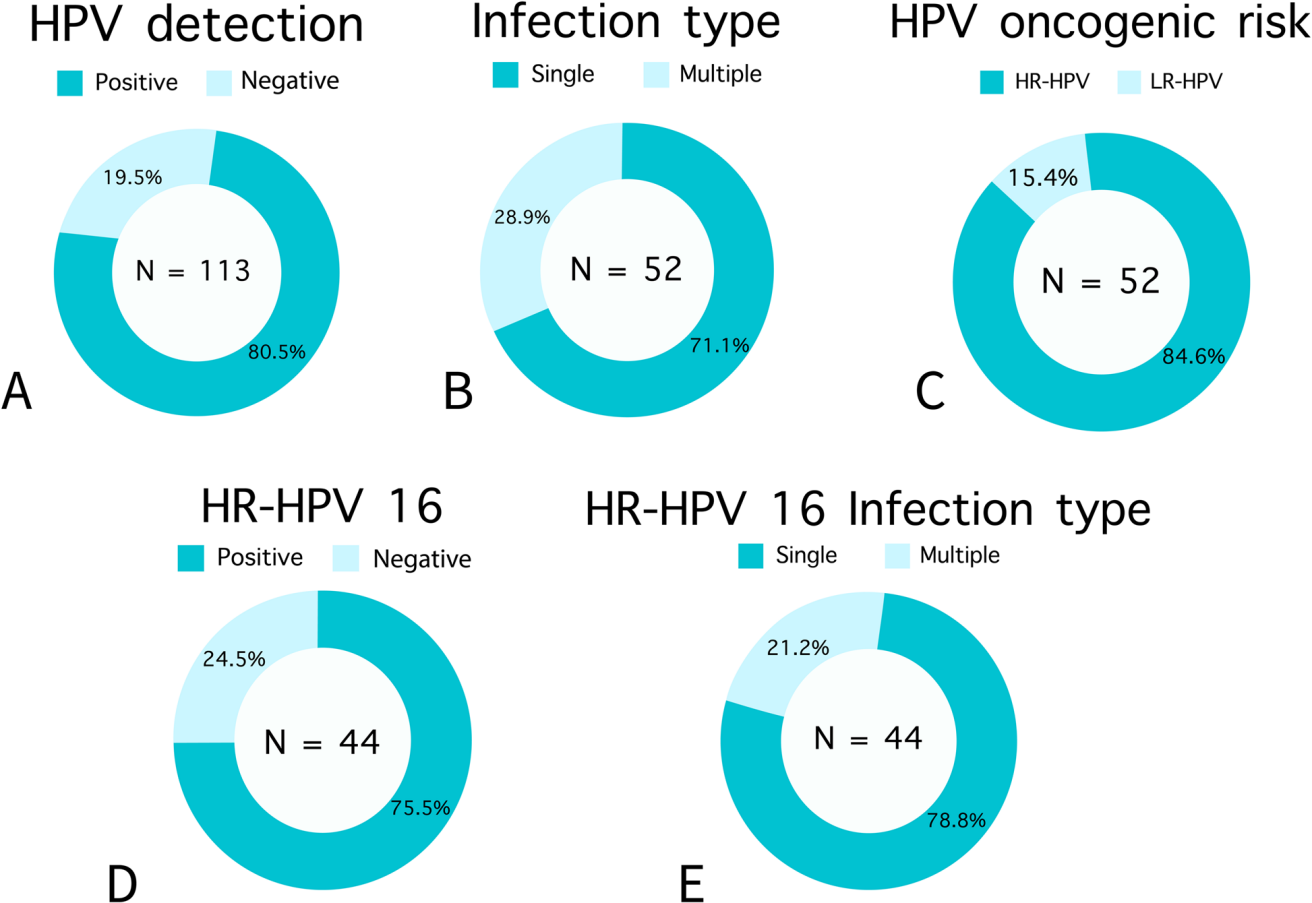
Human papillomavirus (HPV) detection and genotyping data. **A** Global frequency of HPV in penile cancer; **B** Viral type of infection present in positive cases; **C** Separation of positive cases according to the oncogenic risk genotype detected; **D** Frequency of HR-HPV16 infection in positive cases; **E** Type of infection in HR-HPV16-positive cases

In Fig. 3 we describe the main findings of HPV detection and genotyping, showing that 80.5% (91/113) of the tumors were positive for HPV. Determination of the viral type (genotyping) was possible in only 57.1% of the positive cases (52/91). The HR-HPV genotypes detected were 16, 35, 51, 52, 53, 56, 58, 59, 66, and 73; and LR-HPV (low-risk human papillomavirus) were 6, 11, 30, 44, 70, and 74. Type 16 was the most prevalent (75% of positive cases).

HPV positivity was associated with tumors located in the glans (*p* = 0.042) and the presence of carcinoma in situ associated (*p* = 0.029), while the absence of HPV was associated with tumors in advanced stages (pT3-pT4) (*p* = 0.042).

### p16^INK4a^, p53, and ki-67 Protein Expression

Protein expression analysis of p16^INK4a^, p53, and ki-67 by IHC was performed in 173 cases. About 26.0% of cases (45/173) overexpressed p16^INK4a^ and 74.0% (128/173) were negative. The p16^INK4a^ overexpression (positive, score 3) were associated with basaloid (*p* < 0.0001), poorly differentiated (*p* = 0.008), and high proliferative tumors (high ki-67, *p* < 0.0001). Furthermore, the absence of p16^INK4a^ overexpression was associated with tumors without angiolymphatic invasion (*p* = 0.031). The full description of the statistical analyses for koilocytosis, molecular detection of HPV, and p16^INK4a^ expression is described in the supplementary material (S2).

For p53, 55.0% of the cases were negative (95/173), and 45.0% were positive. Among the positive cases, 56.4% were ( +) (44/78) and 43.6% were (+ +) (34/78). The absence of p53 expression was associated with warty tumors (*p* = 0.001), presence of carcinoma in situ (*p* = 0.0.41), HPV-associated histology (*p* < 0.0001), absence of perineural invasion (*p* = 0.036), koilocytosis (*p* = 0.002), and tumors with an expansive growth pattern (*p* = 0.002). In contrast, p53 positivity was associated with poorly differentiated tumors (*p* < 0.0001), pT3-pT4 tumors (*p* = 0.031), stage II disease (*p* = 0.020), and lymph node metastasis (*p* = 0.046).

For ki-67, 26.6% of the cases had high expression (46/173), and 73.4% had low expression (127/173). When analyzing only the hotspot zones, 57.8% of tumors showed high expression (100/173), and 42.2% exhibited low ki-67 expression (73/173). The high expression of ki-67, either global or in hotspot, was associated with basaloid tumors (*p* < 0.0001), poorly differentiated tumors (*p* < 0.0001), presence of angiolymphatic (global *p* = 0.001; hotspot *p* = 0.011), and perineural invasion (global *p* = 0.049; hotspot *p* < 0.0001), pT3-pT4 tumors (global *p* = 0.031; hotspot *p* = 0.014), stage III-IV disease (global *p* = 0.038; hotspot *p* = 0.002), and lymph node metastasis (global *p* = 0.004; hotspot *p* < 0.0001).

In contrast, low ki-67 expression was associated with warty tumors (*p* = 0.017), with an absence of sarcomatoid transformation (global *p* = 0.006; hotspot *p* = 0.001), an expansive invasion pattern (global *p* = 0.001; hotspot *p* < 0.0001), small tumors (global *p* = 0.034), negativity for p53 (global *p* = 0.013; hotspot *p* = 0.002), and with no p16^INK4a^ overexpression (global *p* < 0.0001; hotspot *p* < 0.0001).

**Fig. 4:**
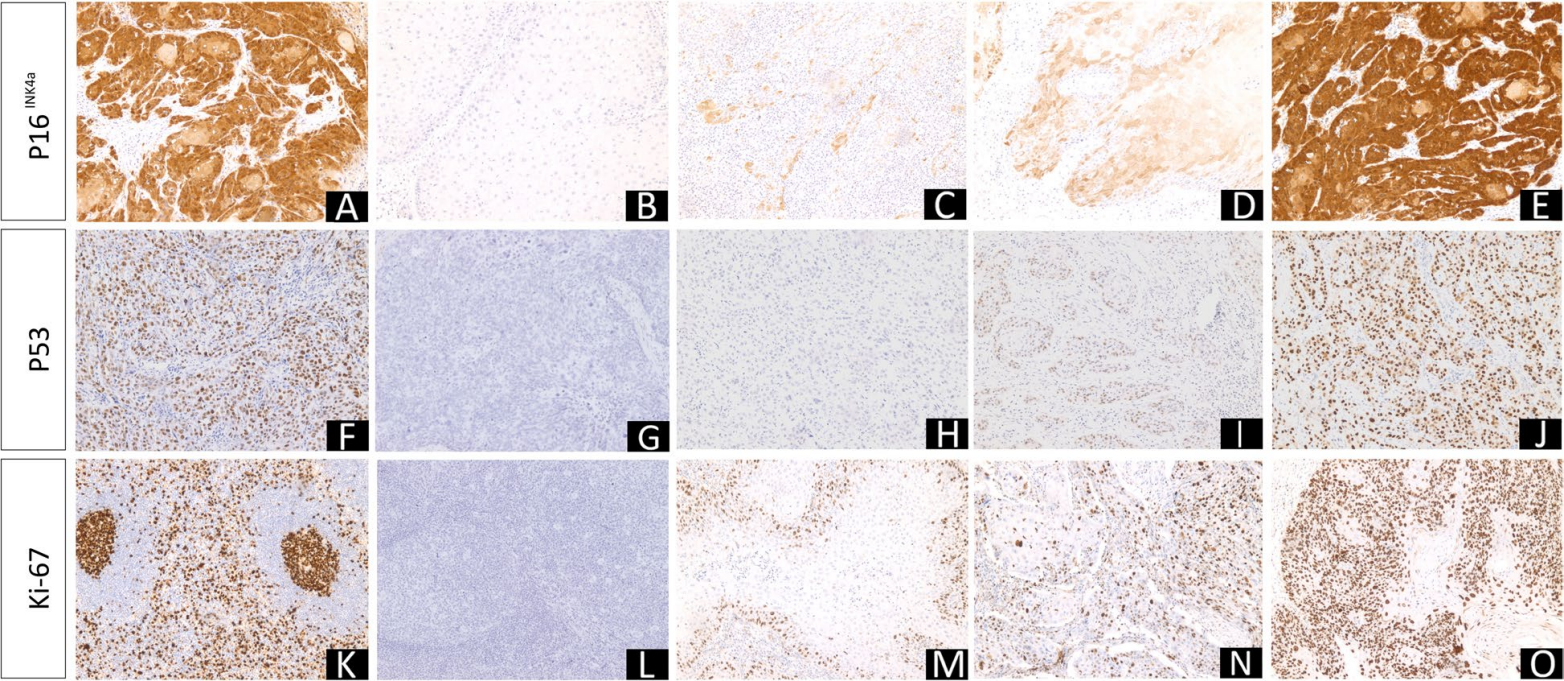
Immunostaining patterns of p16^INK4a^, p53, and ki-67. **A** p16^INK4a^ positive control; **B** p16^INK4a^ negative control; **C** penile cancer showing score 1 for p16^INK4a^; **D** Score 2 for p16^INK4a^; **E** Score 3 for p16^INK4a^; **F** p53 positive control; **G** p53 negative control; **H** PC showing no p53 expression; **I** 40% of tumor cells immunostained for p53; **J** 90% of tumor cells immunostained for p53; (**K**) tonsil used as ki-67 positive control; **L** tonsil with no ki-67 antibody applied and used as the negative control; **M** 20% of cells immunostained for ki-67; **N** 40% of cells immunostained for ki-67; **O** 90% of cells immunostained for ki-67. Magnification 100x (**A**-**O**)

Figure 4 shows the immunostaining profiles for each marker analyzed (p16^INK4a^, p53, and ki-67, and a full description of the statistical analyses for p53 and ki-67 is described in the supplementary material (S3).

### Disease-free survival analysis

For the DFS analysis, we obtained survival data for 134 (67.0%) of the 200 cases evaluated. Approximately 66 patients (33.0%) were excluded because they did not have enough information in their medical records for follow-up analysis or were lost to follow-up. The follow-up time ranged from 1.0 to 114 months (mean 20.96 + 21.32). By the end, 53 cases (53/134) had local recurrence and/or lymph node metastasis (39.5%), and 81 cases (81/134) had no evidence of the disease after surgical treatment (60.5%).

**Fig. 5:**
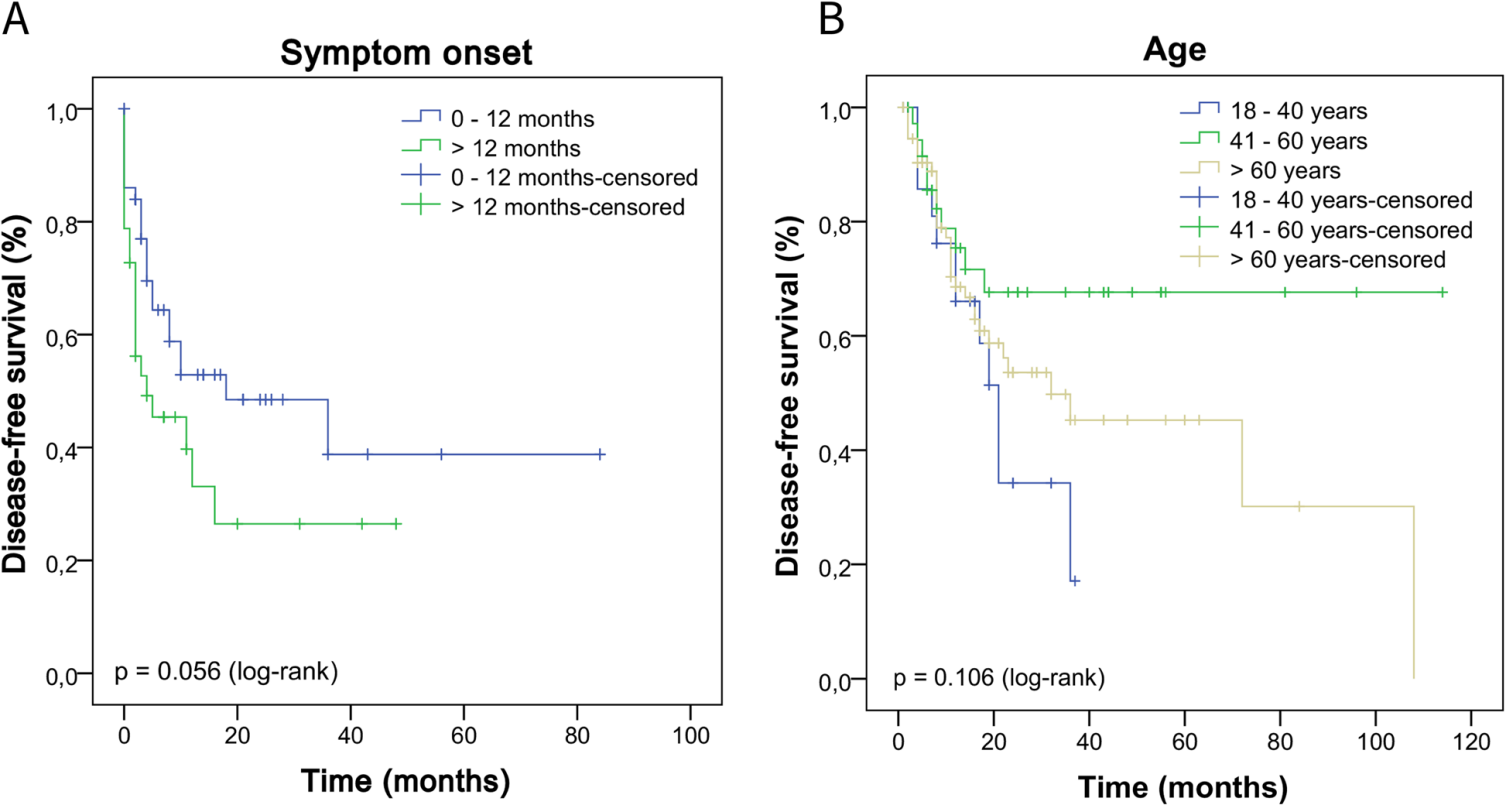
Association of disease-free survival with the delay between the onset of symptoms and diagnosis by patients with PC in Maranhão, Brazil

**Fig. 6:**
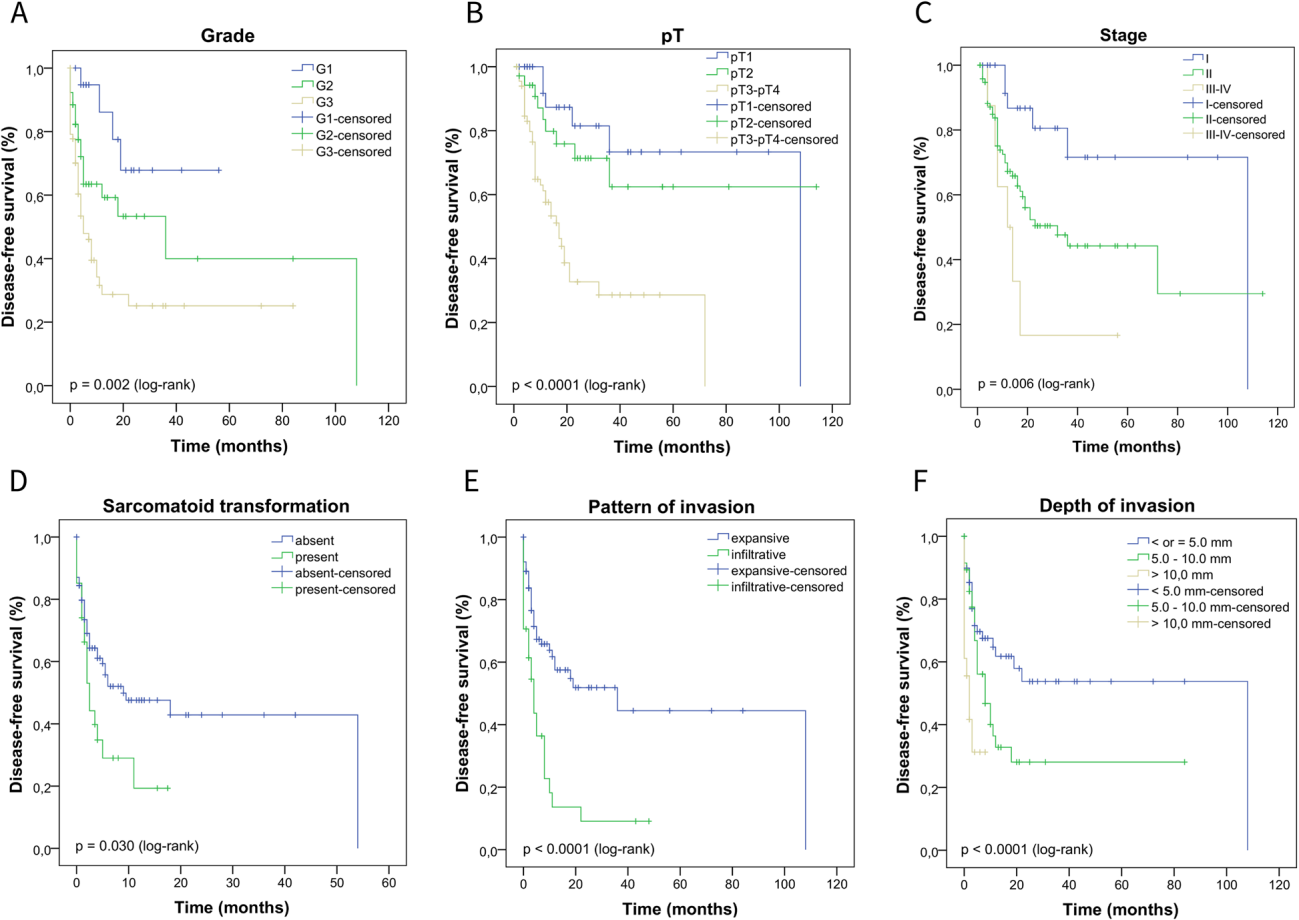
Disease-free survival according to histological features. **A** grade; **B** primary tumor (pT); **C** stage; **D** presence of sarcomatoid transformation; **E** pattern of invasion and **F** depth of invasion

**Fig. 7:**
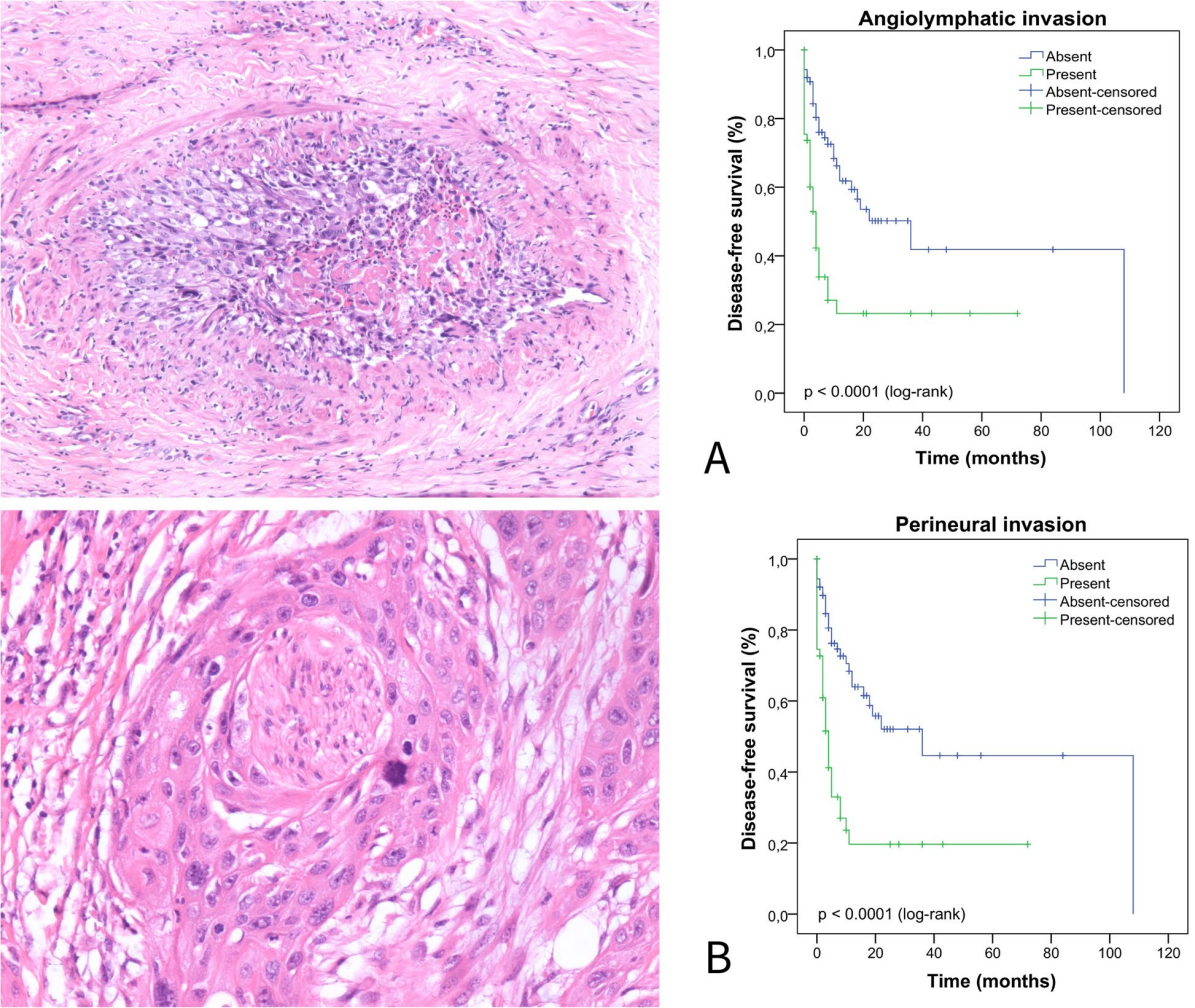
Association of disease-free survival (DFS) with the presence of angiolymphatic and perineural invasion. **A** left, tumor vascular involvement; right, its impact on the DFS of patients with PC; **B** left, tumor perineural involvement; right, its impact on the DFS of patients with PC. Magnification 400x (**A**-**B**)

**Fig. 8:**
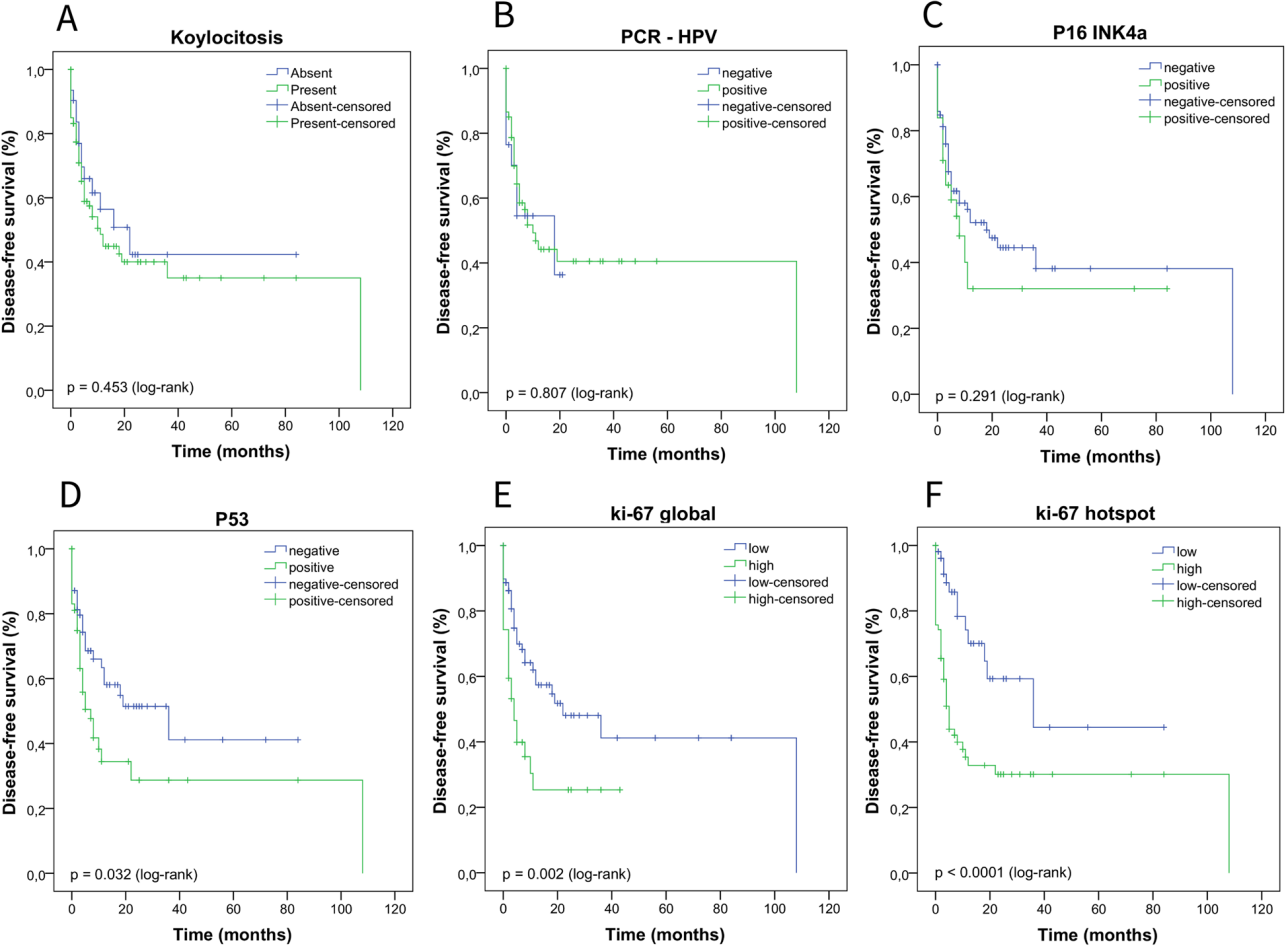
Association of disease-free survival with **A** the presence of koilocytes; **B** HPV molecular detection; **C** expression of p16^INK4a^; **D** p53 expression; **E** global expression of ki-67; and **F** expression of ki-67 in hotspot

We performed the Kaplan–Meier test to estimate the DFS and the log-rank test to compare the survival curves according to clinical, histopathological, HPV infection parameters (koilocytosis, molecular detection, and p16^INK4a^), and p53 and ki-67 protein expression. In Fig. 5, we describe the impact of a delayed diagnosis (log-rank *p* = 0.056) and age on the DFS of these patients (log-rank *p* = 0.106), however, we did not observe a statistically significant difference. In Fig. 6 (A-F), we show the DFS curves according to (A) degree of differentiation (grade), (B) primary tumor (pT), (C) stage, (D) sarcomatoid transformation, (E) pattern of invasion, and (F) depth of invasion. In Fig. 7, we describe PCs with angiolymphatic and perineural invasion and their respective DFS. We observed a statistically significant difference between the survival curves for all parameters shown in Figs. 6 and 7. In Fig. 8 (A-F), we describe DFS according to the presence of koilocytes, molecular detection of HPV, and protein expression of p16^INK4a^, p53, and ki67 (global and hotspot), where we observed a statistically significant difference only with p53 (log-rank *p* = 0.032) and ki-67 expression (log-rank global *p* = 0.002; hotspot *p* < 0.0001).

## Discussion

### Recurrent Profile of PC in Maranhão, Brazil

PC is a neglected and aggressive disease with a sizable impact on quality of life [20]. Its incidence is high in developing countries such as Brazil, particularly in the North and Northeast regions, representing a serious local public health problem [1, 8]. In 2018, our research group found that the state of Maranhão in Northeast Brazil has the highest incidence of PC ever recorded in the world [8]. In 2020, we characterized the sociodemographic features of patients with PC in the region [7].

In this study, we evaluated 200 PC cases diagnosed in Maranhão, integrating clinical-histopathological, immunohistochemical, and HPV infection for a better understanding of disease etiology. The cases were mostly composed of men in their 60 s, the age at which the risk of developing PC is higher; however, it still frequently occurs in younger men [1, 9, 21, 22].

Men under 60 years old accounted for 42.3% of all cases evaluated, with the youngest being only 23 years old. In two studies previously published by our group, 19.7% and 22% of the men were less than 40 years old, respectively [7, 8]. In the present study, we observed a decrease in the survival time of younger individuals (18–40 years old) in relation to those over 40 years old. According to the literature, younger men seem to develop mostly HPV-associated PC, being more prone to relapse, infiltrative patterns, and perineural invasion [23, 24].

We note the prevalence of individuals from the North of Maranhão, where the reference centers are located (in the capital, São Luís). The southern region seems to account for the lowest percentage of cases and this may be associated with the evasion of these patients to cities in neighboring states, such as Piauí (closer than to the capital), or to the southern region of Brazil (looking for better therapeutics approaches). The large migration of PC patients from poor regions to underdeveloped areas is commonly reported in Brazil [1], suggesting that the incidence of PC in Maranhão is possibly even higher than that described by Coelho et al. (2018) [8].

Socioeconomic vulnerability seems to be one of the main risk factors for PC [25]. In the present study, the high prevalence of the disease in men with low education, those with a family income less than a Brazilian minimum monthly wage, and residents/workers from rural areas, reinforce this profile. However, it is important to remember that knowledge about PC and its risk factors seems to be scarce, even among highly educated men [26].

Exploring the profile of these patients, we observed that most of them reported poor genital hygiene habits, an important and known risk factor for PC [27]. Proper penile hygiene has been one of the main prophylactic measures for PC, and when not performed, it favors the emergence of a favorable microenvironment for the proliferation of uropathogenic microorganisms, such as HPV [28]. In addition, it increases the chances of the onset of episodes of chronic inflammation (balanitis and lichen sclerosus), which may also contribute to PC development [29–31].

In addition to the level of education, phimosis also seems to contribute to inadequate cleaning of the penis due to the difficulty or inability in exposing the glans. This condition was described in 70.5% of the cases evaluated in our study and is frequently reported in PC patients. In general, the prevalence of phimosis in PC is approximately 25% to 75%, [32] while it occurs only in 3.4% of healthy adult men [33]. Some studies suggest that phimosis increases the risk of developing PC by 3.5 times, with no protective effect if dealt with after the neonatal stage [3, 34, 35].

Furthermore, we observed a high frequency of risky sexual behavior (54.1%), which can significantly contribute to the acquisition of sexually transmitted infections, such as HPV [36, 37]. A high prevalence (58.7%) of patients who had sex with animals (zoophilia) was shocking data but had been reported before. Vieira et al. (2020) described the practice of zoophilia in 60% of the cases in Maranhão [7]. Both our data and those published by Vieira et al. (2020) corroborate the findings of Zequi et al. (2012), who demonstrated via univariate and multivariate analyses that zoophilia is an important risk factor for PC, but does not seem to impact pathological and clinical prognoses [38].

Upon evaluating the primary symptoms of these patients, we found that the medical records mainly described the appearance of a nodule/tumor in the penis (46.5%) or a foul-smelling ulcerative wound (21.0%). These symptoms are consistent with the main manifestations of the disease, which include changes in skin thickness or color, a wound not having healed in four weeks, rash, bleeding from the penis or under the foreskin, and a smelly odor [39]. Vieira et al. (2020) described the presence of pruritus as the primary initial symptom in patients with PC in Maranhão (26.6%) and discussed its association with lichen sclerosus, despite only 6.0% of cases having this condition in their study [7]. Interestingly, in our study, 27.3% of the cases had associated lichen sclerosus, and the presence of pruritus was observed in 20.2% of the cases, corroborating the findings of Nasca et al. (1999) [40] and Vieira et al. (2020) [7].

As the penis is an external organ, the PC symptoms are classic and easily recognizable. However, we noted an average delay of 17 months between the onset of symptoms and seeking medical care, similar to what was previously described in Maranhão by Vieira et al. (2020), who described a delay of 18.9 months [7]. Late diagnosis is generally attributed to a lack of knowledge about the symptoms of the disease, associated stigmas, and fear of seeking medical help [41]. These factors are intensified in Maranhão, where a large part of the population is socioeconomically vulnerable [42]. The region has the second lowest Human Development Index (HDI) in Brazil (0.639), has rural socioeconomic characteristics, and has historically been marked by significant social inequality and extreme poverty [42].

In this context, access to basic health, especially in rural areas, is scarce, making it difficult to carry out follow-up and early diagnosis of several diseases, including PC. All these factors contribute to an advanced stage at diagnosis, a high rate of metastasis, limitations in the therapeutic approach, and a low overall survival rate. Population-based analyses from Europe and the United States have shown that, unlike other types of tumors, tumor-specific survival rates for PC have not shown any improvement since 1990 [43, 44].

Our findings reinforce a recurrent social-economic profile of patients with PC [7, 8, 45, 46] and call attention to an alarming situation of this disease in poor regions, especially in Maranhão [7, 8, 47]. We suggest that the sum of factors that simultaneously favor disease onsets, such as late diagnosis and advanced stages, may contribute significantly to the high incidence of PC in Maranhão, Brazil, and public policies aimed at remedying these factors in the region are urgent.

### High frequency of HPV-associated PC in Maranhão: differences in disease behavior

The high prevalence of HPV-positive tumors reported in the last five years has been one of the most notable findings in Maranhão. The first study on the detection and genotyping of HPV in local cases was conducted by De Sousa et al. (2015) [48] and Martins et al. (2018) [9], where HPV was reported in 63.15% and 89.1% of tumors analyzed, respectively. Recently, another study described the presence of HPV in 96.4% of cases, the highest frequency ever recorded for PC [13].

All these studies carried out in the region showed a frequency of HPV-positive tumors above the global average of approximately 46.9—50.8% [49–51], and describe HR-HPV 16 as the most common viral type associated with PC. To better understand this situation, in the present study, we used morphological, immunohistochemical, and molecular criteria to investigate the involvement of HPV in the etiology of PC. Based on this, when we performed the molecular detection of HPV, we found a global frequency of HPV infection in 80.5% of PCs, mainly by HR-HPV (84.5%), especially type 16 (75%). We found an association between global HPV positivity and tumors located in the glans, those with carcinoma in situ, and pT1. In fact, glans has been reported as a frequent site of penile HPV infection (35.8%) and is more likely to be infected with multiple HPV types than other sites [52].

In DFS analysis, we did not observe any difference in survival curves according to the HPV status. In contrast, in a cohort from Maranhão, Martins et al. (2018) found better DFS in HPV-positive PC patients than in HPV-negative PC patients [9]; this result has already been reported for PC and other HPV-associated tumors, such as the head and neck [53–55]. Glegoire et al. (1995) demonstrated an association between HPV positivity and worse prognostic factors, such as high staging and aggressive growth patterns [56]; Wiener et al. (1992) found that the overall survival and DFS did not differ between groups in Kaplan–Meier analysis [57].

Histopathological analysis revealed morphological alterations suggestive of HPV infection (koilocytosis) in 78.5% of the evaluated cases. This corroborates previous findings in Maranhão, where koilocytosis was observed in 74.5% of the PC analyzed [9]. The presence of koilocytosis was associated with men reporting more than 10 lifetime sexual partners, tumors with HPV-associated histology, and warty subtype. These findings illuminate the pathophysiology of HPV-associated PC and that in men with risky sexual behavior [58].

Koifman et al. (2011) observed koilocytosis in 88.4% of PC cases evaluated, with high specificity (90%), but low sensitivity in detecting the virus [59]. De-Paula et al. (2007) observed the presence of koilocytosis in 63.1% of the PC samples evaluated, being associated with tumors with low or moderate staging and better DFS [60]. In Maranhão, Nascimento et al. (2020) described angiolymphatic invasion and absence of koilocytosis as predictive factors for lymph node metastasis [10], corroborating previous findings by Ornellas et al. (2008) [61]. However, in the present study, we found no difference in DFS between the groups with and without koilocytosis, as well as no association between koilocytosis and lymph node metastasis.

The presence of koilocytosis is well accepted as pathognomonic evidence for possible the presence of HPV, and has been widely used in screening for cases of viral infection [62]. However, koilocytes are not always present; therefore, it is necessary to use more sensitive and specific methods that also provide information about the viral type and oncogenic risk [63].

To better understand aspects related to HPV infection, we searched the literature for data on the global prevalence of the virus in the general population. In Brazil, the prevalence of HPV in individuals without clinical symptoms appears to vary from 30 to 75%, depending on the region studied [64]. Interestingly, Maranhão represents one of the highest rates recorded in the country (59.1%), according to sampling carried out in the capital (São Luís) [65]. It is believed that in the interior regions of the state, where most PC patients come from, these rates may be even higher, but there are no studies with a representative sample of these locations.

The high prevalence of healthy individuals infected with HPV in Maranhão has been attributed to factors such as early initiation of sexual activity (average age, 15.5) and the high number of sexual partners [65]. In the present study, we did not assess the onset of sexual activity, but we observed a statistically significant association between risky sexual behavior and the presence of koilocytosis in PC patients, which corroborates the epidemiological HPV aspects of this disease.

Knowledge of the regional prevalence of HPV infection is important for understanding the local etiology of PC, as the incidence of HPV-associated tumors seems to be proportional to the prevalence of viral infection in healthy individuals [66]. In our series, when we searched for tumors that were negative for koilocytosis, HPV infection, and p16^INK4a^ overexpression, only one case was not positive for any of these three parameters. Interestingly, this was the first case report of pseudoangiosarcomatous squamous cell carcinoma on the penis, an uncommon and highly aggressive variant of squamous cell carcinoma [67].

In addition to koilocyte and HPV molecular detection, we performed p16^INK4a^ protein expression analysis, which is widely used as an indirect marker of HR-HPV infection and has been associated with basaloid and high-grade tumors [9, 68, 69]. However, the absence of p16^INK4a^ overexpression does not exclude infection by LR-HPV or even the absence of HPV infection [17, 70].

We found p16^INK4a^ overexpression in 26.0% of PC cases. In the present study, all basaloid tumors showed p16^INK4a^ overexpression, reinforcing its secondary use as a tool in the differential diagnosis of this histological subtype [9, 17, 70]. Furthermore, the lack of p16^INK4a^ overexpression was associated with the absence of angiolymphatic invasion.

According to Cubilla et al. (2011), p16^INK4a^ overexpression is closely correlated with the presence of the HR-HPV genotype in PC, as observed in cervical carcinoma and other HPV-related tumors [17, 70]. However, in the present study, no association between p16^INK4a^ overexpression and HR-HPV infection was observed. We suggest that limitations in HPV detection methods may explain these findings, such as: (1) we used DNA samples obtained from FFPE tissue, unlike Martins et al. (2018), who used fresh tumor samples; and (2) viral genotyping was not done for all positive cases.

Despite the high frequency of cases with koilocytes and HPV-positivity, the frequency of p16^INK4a^ overexpression was low compared to that described by Martins et al. (2018) in Maranhão, where 40% of the cases had overexpression of p16^INK4a^ [9]. We speculate that the use of different antibody clones for IHC of p16^INK4a^ might underlie this difference. Martins et al. (2018) described four basaloid tumors in a total of 55 cases, while here we had eight basaloid tumors in approximately 173 cases.

We also did not observe a statistically significant difference in DFS with the level of p16^INK4a^ expression. However, patients with p16^INK4a^overexpression lived on average less (30.1 months) when compared to patients without p16^INK4a^ overexpression (46.8 months). These findings do not corroborate with previous studies where PC men with HPV or p16^INK4a^ overexpression generally have a significantly more favorable DFS compared to those without HPV or p16^INK4a^ overexpression [71]. Furthermore, a recent meta-analysis showed that p16^INK4a^ overexpression was significantly associated with and was an independent factor for better DFS in PC patients (HR = 0.45, 95% CI: 0.30–0.67, *P* < 0.001) [72].

Given these controversial data, we performed DFS analysis for p16^INK4a^ overexpression according to the following criteria: (1) basaloid tumors were excluded; and only (2) HR-HPV positive cases; (3) usual subtype tumors; (4) warty subtype tumors; and (5) stage I-II and III-IV tumors. However, in all analyses, there was no significant difference in DFS, and the mean survival time of the p16^INK4a^ overexpressing group remained lower than that of no overexpressed p16^INK4a^.

Unfortunately, Martins et al. (2018) did not describe survival data based on p16^INK4a^ expression, making it difficult to compare their findings in Maranhão with those observed in the present study [9]. However, similar to what was observed in our study, in a study conducted in Northern Brazil (state of Amazonas), no differences were observed between the survival curves of PC patients with p16^INK4a^ overexpression (log-rank *p* = 0.753) or molecular HPV positivity (log-rank *p* = 0.979) [73]. According to Ornellas & Ornellas (2018), it is uncertain whether PCs involving HPV infection have better survival profiles than those without HPV infection [74]. Our data and those published in Amazonas suggest a different impact of p16^INK4a^ overexpression in PC patients from these Brazilian regions.

For histopathological classification, all the evaluated tumors were classified as squamous cell carcinoma (SCC), the most common type of PC [23]. According to the WHO [75], these tumors are grouped into HPV-associated subtypes (basaloid, papillary-basaloid, warty, warty-basaloid, and clear cell carcinoma) and non-associated HPV (usual, pseudohyperplastic, pseudoglandular, verrucous pure or cuniculatum, papillary, adenosquamous, and sarcomatoid).

In the present study, most cases were the usual subtype (39.5%), followed by the warty (29.8%). These frequencies differ from other studies in the area, such as that carried out by Cubilla et al. (2001), where out of 61 tumors evaluated, 59% corresponded to the usual subtype and only 10% corresponded to warty tumors [76]. In 2010, Chaux et al. compared the distribution of different histological subtypes of PC from regions of high (Paraguay, 144 cases) and low (USA, 157 cases) incidence and observed that the prevalence of histological subtypes in the Paraguayan and American series was as follow: usual, 49.3% and 46.5%; verrucous, 8.3% and 7.6%; papillary NOS, 7.6% and 5.7%; warty, 6.9% and 8.3%; basaloid, 4.2% and 7.0%; sarcomatoid, 0.7% and 0.6%; adenosquamous, 3.5% and 0.6%; and mixed, 19.4% and 23.6%, respectively [77].

In 2018, Martins et al. observed that 47.3% of the PC cases evaluated in Maranhão were usual and 29.1% were warty subtypes [9]. Subsequently, Vieira et al. observed 40.0% of usual tumors and 33.0% of warty tumors [7]. Another important aspect is the high frequency of mixed tumors (with characteristics of more than one histological subtype), which in the present study was approximately 32.0%, higher than that described by Martins et al. 2018 (16.3%) and Vieira et al. 2020 (25.0%). Considering mixed tumors according to the presence or absence of an HPV-associated component, similar to the classification described by Canete-Portillo et al. (2020) [78], all mixed tumors in the present study had a basaloid, warty-basaloid, or warty component associated with another component (usual). Thus, the overall prevalence of HPV-associated histology in the cases evaluated in our study go up to 77.5%.

The high frequency of warty tumors in the present study and in others carried out in Maranhão, as well as the prevalence of mixed tumors with HPV subtypes associated, differs drastically from that observed in other studies, and supports the high prevalence of HPV-associated PC in the region [76, 77].

### Expression of p53 is Related to Worse Prognosis and Lymph node metastasis

When we evaluated the p53 expression profile, negative cases were more prevalent (55.0%) than positive cases (45.0%). The statistical analysis revealed that p53 positivity was associated with worse prognostic factors such as high-grade tumors (G3), pT3-pT4, those in stage II, and those with lymph node metastasis, corroborating previous studies with PC and other tumor types [79–81].

Previously, all poorly differentiated PCs analyzed by Marinescu et al. (2016) were p53 positive [82], and Lopes et al. (2002) found that p53 expression was an independent factor for lymph node metastasis (relative risk 4.8, 95% CI 1.6 to 14.9); the 5 and 10-year overall survival rates were 65.2% and 54.6% in PCs with negative p53, and 30.2% and 26.4%, respectively, in those with positive p53 [18]. In this study, p53 expression was associated with lower DFS when compared to negative cases.

According to the DGIdb database, the TP53 gene is the target of 193 therapeutic drugs [83], many of which are already used for head and neck tumors, which appear to have a mutational signature very similar to PC [84]. Overexpression of p53 is frequently associated with disruptive mutations and oncogenic activation in HPV-negative PC, possibly in cooperation with other mutations in genes of the HER-PTEN-AKT pathway [85].

In a study conducted by Jacob et al. (2019), most patients with metastatic PC had mutations in TP53 and were HPV-negative [86] This suggests that TP53 mutations appear to be the main factor associated with metastasis in patients with advanced PC, whereas HPV-related oncogenic mechanisms appear to be more effective in the early stages of the disease and are gradually lost [85, 86]. These findings may explain the pathophysiological differences observed in this study and in other studies carried out in Maranhão, where most cases are diagnosed in the advanced stages of the disease.

In contrast to what we observed for p53 positivity, the absence of p53 expression was associated with warty tumors, presence of carcinoma in situ, tumors with HPV-associated histology, absence of perineural invasion, koilocytosis, and expansive growth pattern. These findings corroborate the main pathway for HPV action, where the viral oncoprotein E6 binds to the p53 protein and inhibits its tumor suppressor function [87]. In addition, a previous study in Maranhão found that 81.0% of PC cases were negative for p53 expression by IHC and 83.3% presented TP53 gene downregulation [13].

### Ki-67 Expression as a Marker of Tumor Aggressiveness in PC, Including Perineural and Angiolymphatic Invasion

Most tumors analyzed in the present study had a low global expression of ki-67 (73.4%), but high expression in hotspot zones (57.8%). Here, we observed an association between high ki-67 expression and basaloid and high-grade tumors, presence of angiolymphatic and perineural invasion, pT3-pT4 tumors, stage III-IV, and lymph node metastasis. The association between high expression of ki-67 and worse prognostic factors has been previously described in PC [19, 88, 89].

Ki-67 analysis has been used as a good parameter to assess the proliferative index and aggressiveness of numerous tumors [90, 91]. In PC, Stankiewicz et al. (2012) found an association between high expression of ki-67, basaloid tumors, and HPV infection, corroborating the results of the present study, in which all basaloid tumors had high ki-67 expression [89]. In contrast, we observed that warty tumors were associated with low ki-67 expression (global and hotspot). This difference corroborates the clinical behavior of each histological subtype, where basaloid tumors generally have fast growth and generally poor prognosis, while warty tumors have slow growth and are more often a good prognosis [88].

A strong association between high ki-67 expression and poorly differentiated tumors has been described previously in PC [89]. Berdjis et al. (2005) observed an inverse relationship between ki-67 expression and tumor differentiation (*p* < 0.0005), but they did not observe a relationship with advanced staging or lymph node metastasis, unlike our findings [19].

The association between high ki-67 expression and angiolymphatic and perineural invasion is another important finding of our study and was previously described for PC. Angiolymphatic and perineural invasions are known to be associated with a worse prognosis, and its detection is sometimes influenced by the observer (pathologist) bias, suggesting that IHC for ki-67 can refine the diagnosis and prompt one to search for neural or vascular invasion in the histopathological analysis [92, 93].

Protzel et al. (2007) and Zhu et al. (2007) described a strong association between high ki-67 expression and lymph node metastasis in PC [79, 88], and Warli et al. (2020) argued that this association appears to be independent of grade and stage [94]. Our findings also demonstrated an association between high ki-67 expression and the occurrence of lymph node metastasis in PC patients, indicating the role of ki-67 as a predictive factor for metastasis in these patients.

Despite the association with worse prognostic factors and lymph node metastasis, we did not observe a statistically significant difference between the DFS rate according to the ki-67 expression profile, similar to the study published by Stankiewicz et al. (2012) [89]. We hypothesized that the cumulative effect of other factors such as staging, angiolymphatic invasion, and perineural invasion in our study may have influenced DFS patients more than ki-67 high expression. For our studies, future multivariate analyses are required to elucidate these aspects. However, high ki-67 expression has been reported to be associated with worse DFS in PC (log-rank p = 0.0098) [88].

### Considerations and perspectives

PC is a neglected, heterogeneous, aggressive, and deadly disease that is typically not diagnosed early. There is a high incidence of PC in the Maranhão state, and little is known about the local etiological profile of these tumors. Most patients are socioeconomically vulnerable and come from the state's countryside. There are few centers for treating the disease in the region, most of which are located in the capital, São Luís, making early diagnosis and monitoring of these patients challenging.

Here, we discussed the impact of the geographic isolation of the state capital on the displacement of these patients, suggesting that the actual incidence of the disease is more significant than that described previously [8]. Our findings reinforce a profile of the disease in the state consistent with other studies and bring new insights into possible factors that, together, contribute to Maranhão having the highest incidence of PC in the world, such as the long delay in diagnosis and the high prevalence of HPV infection.

According to Ornellas & Ornellas (2018), when PC is considered globally, only a tiny proportion is described as HPV-associated. Therefore, increased men's education and prevention strategies such as condom use, hygienic measures, smoking cessation, and avoidance of chronic inflammatory states can considerably impact the pathogenesis of precancerous lesions of the penis [74]. In Maranhão, where a significant number of PCs appear to be HPV-associated and most patients are socioeconomically vulnerable, both HPV immunization and patient education could impact disease rates.

Our data discuss HPV infection in a multi-methodological way, integrating histological, molecular, and immunohistochemical aspects and bringing new insights into the role of HPV on histological presentation and clinical behavior of these tumors. An interesting question involves the histological and prognostic variation observed in HPV-associated tumors. For example, why do warty and basaloid tumors, both HPV-associated, have widely different prognoses? Why does p16^INK4a^ overexpression appear to be related to decreased disease-free survival and what is its impact on overall and cancer-specific survival in PC patients? To answer these questions we need to explore the correlation between *CDKN2a* (p16^INK4a^) copy number, mRNA expression, methylation profile, and protein expression (IHC).

Another important approach that we are currently investigating is the biological profile of viral oncoproteins (E6 and E7), both by gene (mRNA) and protein expression. These data linked to the characterization of p16^INK4a^ can reveal the real status of HPV infection in these patients, whether it is active and impacting tumor progression or part of the HPV-positive cases are just a background due to the high rate of HPV infection in the general population, especially in Maranhão (as discussed).

The joint analysis of this information can help to better characterize each of the cited markers and their real role in the patient's prognosis. The perspective of our group has been, given the vast majority of HPV-positive cases, to investigate the differences within this group. We are currently performing all these analyzes on a prospective cohort of 60 cases from Maranhão, involving histological (H&E, IHC), genomic (exome, RNA-seq, miRNA-seq), and metagenomic tools to unravel these questions. All these approaches ongoing rely on the expertise of geneticists, physicians, pathologists, and oncologists with experience in PC to perform an integrative analysis between genomic (multi-omics) and the clinical aspects of PC. We hope with this approach to also elucidate the role of changes in p53 in PC carcinogenesis, evaluating somatic mutations and gene expression patterns.

At the time, the associations found between p16^INK4a^, p53, and ki-67 expression have clinical relevance and seem to impact the DFS of these patients. The test used to assess these protein expression profiles (IHC) is offered free of charge by the Brazilian Unified Health System (SUS) and can be used in histopathological routines to help diagnostic and prognostic classification of these patients.

Despite the difficulties inherent in the PC study, the present study strongly points to a clinical-histopathological and etiological profile different from that observed in other studies in Brazil and worldwide. It highlights the importance of local and collaborative research aimed at understanding these tumors' etiological aspects, behavior, and progression, especially in places with high incidences, such as Brazil. We emphasize that, due to limitations in current protocols for the management of PC patients, early diagnosis becomes even more decisive for better therapeutic conduct. This reality is expected to change since recently, the Brazilian consensus statement for managing PC patients in low- and middle-income countries has been proposed [95].

### Limitations

In the present study, we presented important clinical-histopathological and pathophysiological features of PC. However, some limitations may reduce the clinical impact of the data obtained and must be considered. First, the retrospective aspect of the study makes it difficult to have reliable control over the variables evaluated, creating gaps in the data. Second, the use of DNA from FFPE tumor samples may increase HPV-PCR false negatives and the absence of genotyping data for all HPV-positive tumors limits the stratification of cases according to the HPV genotype. Third, this study had many censored patients in DFS analysis due to loss of follow-up and there is no data regarding cancer-specific and/or overall survival. Despite this, the data obtained from a large Brazilian cohort of PC present a wealth of information that significantly contributes to a better understanding of this neglected disease. All limitations may be further explored in a future prospective study, as discussed.

## Conclusions

Our study obtained important clinical and histopathological data for the characterization of the epidemiological profile of PC in Maranhão, Brazil. Our IHC findings revealed important parameters with prognostic values, including ki-67 and p53 expression associated with lymph node metastasis, grade, pT, stage, pattern, and depth of invasion, as well as ki-67 and p53 expression associated with decreased survival of PC patients. Additionally, p16^INK4a^ overexpression can be used as a marker for basaloid tumors, an HR-HPV-related histological subtype. The IHC is easily accessible in poorer regions such as Brazil, reinforcing its applicability in clinical practice.

Our data reaffirmed the high incidence of human papillomavirus (HPV) infection in PC and highlighted its possible association with worse clinical prognosis factors in the cohort evaluated, differently from what was observed in other regions. The association between HPV histological changes and men with risky sexual behavior draws attention to the urgency of effective public policies to vaccinate men to prevent the disease. This is especially important in Brazil, where there is a high rate of amputations as the first treatment option. Finally, our integrative approach offers new insights into possible factors that together contribute to Maranhão having the highest global incidence of PC ever recorded and highlights the importance of studies that aim to understand the pathophysiological behavior of HPV-related PCs better.

## Supplementary Information

**Additional file 1: Supplementary material (S1) Table 1. **Primers used for HPV detection in penile cancer samples** Table 2. **PCR mix preparation and thermocycling conditions.

**Additional file 2: Supplementary material (S2) Table 1.** Statistical data for koilocytosis, molecular detection of HPV, and p16^INK4a^ expression analysis in penile cancer.

**Additional file 3: Supplementary material (S3) Table 1.** Statistical data for p53 and ki-67 protein expression analysis in penile cancer.

## Data Availability

The data used in this study are not publicly available due to privacy and ethical regulations, being made available only when requested to the corresponding author.

## Abbreviations

PC: Penile cancer
PCR: Polymerase chain reaction
FFPE: Formalin-fixed paraffin embedded
HPV: Human papillomavirus
HR-HPV: High-risk human papillomavirus
HIV: Human immunodeficiency virus
H&E: Hematoxilina e eosina
HU-UFMA: Hospital Universitário da Universidade Federal do Maranhão
HCAB: Hospital do Câncer Aldenora Bello
DFS: Disease-free survival
IHC: Immunohistochemistry
IARC: International Agency for Research on Cancer
ASR: Age-standardized rate
LR-HPV: Low-risk human papillomavirus
