# Supplementary material for: A comprehensive analysis of penile cancer in the region with the highest worldwide incidence reveals new insights into the disease
Source: BMC Cancer. 2022 Oct 15;22:1063. doi: 10.1186/s12885-022-10127-z (PMC9569053; doi:10.1186/s12885-022-10127-z)
Supplement: Supplementary file 1 — Additional file 1: Supplementary material (S1) Table 1. Primers used for HPV detection in penile cancer samples Table 2. PCR mix preparation and thermocycling conditions. [file 12885_2022_10127_MOESM1_ESM.docx]

**Supplementary material (S1)**

**Table 1.** Primers used for HPV detection in penile cancer samples

| Primers set | Primers name | sequence (5’-3’) |
| --- | --- | --- |
| PGMY 09/11 | PGMY11-A | GCA CAG GGA CAT AAC AAT GG |
|  | PGMY11-B | GCG CAG GGC CAC AAT AAT GG |
|  | PGMY11-C | GCA CAG GGA CAT AAT AAT GG |
|  | PGMY11-D | GCC CAG GGC CAC AAC AAT GG |
|  | PGMY11-E | GCT CAG GGT TTA AAC AAT GG |
|  | PGMY09-F | CGT CCC AAA GGA AAC TGA TC |
|  | PGMY09-G | CGA CCT AAA GGA AAC TGA TC |
|  | PGMY09-H | CGT CCA AAA GGA AAC TGA TC |
|  | PGMY09-I | G CCA AGG GGA AAC TGA TC |
|  | PGMY09-J | CGT CCC AAA GGA TAC TGA TC |
|  | PGMY09-K | CGT CCA AGG GGA TAC TGA TC |
|  | PGMY09-L | CGA CCT AAA GGG AAT TGA TC |
|  | PGMY09-M | CGA CCT AGT GGA AAT TGA TC |
|  | PGMY09-N | CGA CCA AGG GGA TAT TGA TC |
|  | PGMY09-P | G CCC AAC GGA AAC TGA TC |
|  | PGMY09-Q | CGA CCC AAG GGA AAC TGG TC |
|  | PGMY09-R | CGT CCT AAA GGA AAC TGG TC |
|  | HMB01 | GCG ACC CAA TGC AAA TTG GT |
| β-globin | GH2O | GAA GAG CCA AGG ACA GGT AC |
|  | PCO4 | CAA CTT CAT CCA CGT TCA CC |
| GP5+/6+ | GP5+ | TTT GTT ACT GTG GTA GAT ACT AC |
|  | GP6+ | GAA AAA TAA ACT GTA AAT CAT ATT C |

Primer sequences used to detect human papillomavirus (HPV) in penile cancer. The PGMY09/11 primer set consists of five forward sequences (PGMY11A-E) and 13 reverse sequences (PGMY09F-HMB01), being used in the first round of PCR. The GP5+/GP6+ primers consist of a simple forward and a reverse sequence, used in the second round of amplification. The GH2O and PCO4 primers are β-globin-targeted sequences and were used as a control for the viability of DNA samples.

**Table 2.** PCR mix preparation and thermocycling conditions

| Primer | Mix (1X) | Cycle conditions* |
| --- | --- | --- |
| β-globin | 12,5 µL of *Master Mix*  1,5 µL of GH2O (10 µM)  1,5 µL of PCO4 (10 µM)  7,5 µL of nuclease-free water  *2,0 µL of DNA (50-100 ng/*µl) | 95°C for 5 min.    95°C for 60s.  **40x** 55°C for 60s.  72°C for 2 min.  72°C for 8 min. |
| PGMY 09/11 | 12,5 µL of *Master Mix*  0,4 µL of PGMY09 (10 µM)  0,4 µL of PGMY11 (10 µM)  9,7 µL of nuclease-free water  *2,0 µL of DNA (50-100 ng/*µl) | 95°C for 5 min.    95°C for 40s.  **40x** 55°C for 40s.  72°C for 40s.  72°C for 8 min. |
| GP5+/6+ | 12,5 µL *Master Mix*  2,5 µL of GP5+ (10 µM)  2,5 µL of GP6+ (10 µM)  2,5 µL of nuclease-free water  *5,0 µL of PGMY product* | 94°C for 5 min.    94°C for 45s.  **40x** 54°C for 60s.  72°C for 40s.  72°C for 8 min. |

The concentration of each use solution for each primer set was 10 µM. For PGMY09/11, the use solution was prepared from a PGMY09F-HMB01 primers pool (10 µM/primer) and a PGMY11A-E primers pool (10 µM/primer); (*) in thermocycling we used “min” for “minutes” and “s” for seconds.
