# Supplementary material for: A comprehensive analysis of penile cancer in the region with the highest worldwide incidence reveals new insights into the disease
Source: BMC Cancer. 2022 Oct 15;22:1063. doi: 10.1186/s12885-022-10127-z (PMC9569053; doi:10.1186/s12885-022-10127-z)
Supplement: Supplementary file 2 — Additional file 2: Supplementary material (S2) Table 1. Statistical data for koilocytosis, molecular detection of HPV, and p16INK4a expression analysis in penile cancer. [file 12885_2022_10127_MOESM2_ESM.docx]

**Supplementary material (S2)**

**Table 1**. Statistical data for koilocytosis, molecular detection of HPV, and p16INK4a expression analysis in penile cancer

| **Parameters** | **Koilocytosis** | | ***p value*** | **p16^INK4a^ overexpression** | | ***p value*** | **HPV-PCR** | | ***p value*** |
| --- | --- | --- | --- | --- | --- | --- | --- | --- | --- |
|  | Absent | Present |  | Negative | Positive |  | Negative | Positive |  |
| ***Age*** |  |  |  |  |  |  |  |  |  |
| 18 – 40 | 9 (20.9) | 18 (11.5) | *0.080* | 14 (11.0) | 10 (22.2) | *0.127* | 4 (18.2) | 16 (17.6) | *0.808* |
| 41 – 60 | 15 (34.5) | 42 (26.9) |  | 37 (29.1) | 14 (31.1) |  | 5 (22.7) | 27 (29.7) |  |
| > 60 | 19 (44.2) | 96 (61.5) |  | 76 (59.8) | 21 (46.7) |  | 13 (59.1) | 48 (52.7) |  |
| ***Schooling*** |  | | | | | | | | |
| No education | 12 (63.2) | 62 (80.5) | *0.103* | 49 (75.4) | 21 (84.0) | *0.882* | 13 (86.7) | 56 (80.0) | *1.000* |
| Primary school | 7 (36.8) | 12 (15.6) |  | 14 (21.5) | 4 (16.0) |  | 2 (13.3) | 12 (17.1) |  |
| Secondary school | 0 (0.0) | 3 (3.9) |  | 2 (3.1) | 0 (0.0) |  | 0 (0.0) | 2 (2.9) |  |
| ***Smoking habits*** |  | | | | | | | | |
| No | 14 (53.8) | 35 (35.0) | *0.113* | 39 (45.9) | 7 (25.9) | *0.076* | 9 (50.0) | 32 (41.6) | *0.601* |
| Yes | 12 (46.2) | 65 (65.0) |  | 46 (54.1) | 20 (74.1) |  | 9 (50.0) | 45 (58.4) |  |
| ***Alcoholism*** |  |  |  |  |  |  |  |  |  |
| No | 10 (43.5) | 29 (45.3) | *1.000* | 29 (50.0) | 7 (36.8) | *0.428* | 7 (53.8) | 22 (42.3) | *0.540* |
| Yes | 13 (56.5) | 35 (54.7) |  | 29 (50.0) | 12 (63.2) |  | 6 (46.2) | 30 (57.7) |  |
| ***Phimosis*** |  |  |  |  |  |  |  |  |  |
| No | 3 (14.3) | 30 (33.0) | *0.114* | 23 (28.8) | 6 (27.3) | *1.000* | 5 (31.2) | 24 (31.6) | *1.000* |
| Yes | 18 (85.7) | 61 (67.0) |  | 57 (71.2) | 16 (72.7) |  | 11 (68.8) | 52 (68.4) |  |
| ***Zoophilia*** |  |  |  |  |  |  |  |  |  |
| No | 6 (50.0) | 20 (39.2) | *0.530* | 20 (42.6) | 6 (46.2) | *1.000* | 6 (50.0) | 18 (41.9) | *0.745* |
| Yes | 6 (50.0) | 31 (60.8) |  | 27 (57.4) | 7 (53.8) |  | 6 (50.0) | 25 (58.1) |  |
| ***Genital hygiene*** |  |  |  |  |  |  |  |  |  |
| Good | 4 (28.6) | 18 (27.7) | *1.000* | 17 (28.3) | 4 (30.8) | *1.000* | 4 (26.7) | 15 (26.8) | *1.000* |
| Poor or moderate | 10 (71.4) | 47 (72.3) |  | 43 (71.7) | 9 (69.2) |  | 11 (73.3) | 41 (73.2) |  |
| ***STD history*** |  |  |  |  |  |  |  |  |  |
| No | 5 (45.5) | 29 (48.3) | *1.000* | 27 (51.9) | 6 (42.9) | *0.764* | 7 (53.8) | 23 (52.3) | *1.000* |
| Yes | 6 (54.5) | 31 (51.7) |  | 25 (48.1) | 8 (87.1) |  | 6 (46.2) | 21 (47.7) |  |
| ***N° sexual partners*** |  |  |  |  |  |  |  |  |  |
| < 6 | 3 (30.0) | 14 (27.5) | *0.001* | 14 (34.4) | 3 (25.0) | *0.913* | 5 (38.5) | 11 (28.2) | *0.197* |
| 6 – 10 | 6 (60.0) | 5 (9.8) |  | 9 (19.6) | 2 (16.7) |  | 0 (0.0) | 9 (23.1) |  |
| > 10 | 1 (10.0) | 32 (62.7) |  | 23 (50.0) | 7 (58.3) |  | 8 (61.5) | 19 (48.7) |  |
| ***Symptoms onset*** |  |  |  |  |  |  |  |  |  |
| 0 – 12 months | 17 (60.7) | 47 (58.8) | *1.000* | 46 (61.3) | 11 (55.0) | *0.617* | 10 (62.5) | 32 (56.1) | *0.778* |
| > 12 months | 11 (39.3) | 33 (41.2) |  | 29 (38.7) | 9 (45.0) |  | 6 (37.5) | 25 (43.9) |  |
| ***Surgical procedure*** |  |  |  |  |  |  |  |  |  |
| Preservative (exeresis) | 3 (7.0) | 4 (2.5) | *0.524* | 5 (3.9) | 0 (0.0) | *0.793* | 0 (0.0) | 0 (0.0) | *0.488* |
| Total glansectomy | 1 (2.3) | 6 (3.8) |  | 6 (4.7) | 1 (2.2) |  | 0 (0.0) | 66 (72.5) |  |
| Partial penectomy | 33 (76.7) | 112 (71.3) |  | 92 (71.9) | 34 (75.6) |  | 14 (63.6) | 13 (14.3) |  |
| Total penectomy | 4 (9.3) | 24 (15.3) |  | 17 (13.3) | 7 (15.6) |  | 6 (27.3) | 8 (8.8) |  |
| Emasculation | 2 (4.7) | 11 (7.0) |  | 8 (6.2) | 3 (6.7) |  | 2 (9.1) | 91 (100.0) |  |
| ***Topography*** |  |  |  |  |  |  |  |  |  |
| Foreskin and/or coronal sulcus | 4 (9.5) | 4 (2.6) | *0.133* | 7 (5.5) | 0 (0.0) | *0.207* | 0 (0.0) | 2 (2.2) | *0.042* |
| Glans | 22 (52.4) | 66 (42.3) |  | 58 (45.7) | 17 (37.8) |  | 6 (27.3) | 35 (38.5) |  |
| Corpus | 2 (4.8) | 5 (3.2) |  | 3 (2.4) | 2 (4.4) |  | 1 (4.5) | 2 (2.2) |  |
| Glans + foreskin and/or coronal sulcus | 7 (16.7) | 47 (30.1) |  | 32 (25.2) | 15 (33.3) |  | 4 (18.2) | 34 (37.4) |  |
| Glans + corpus | 7 (16.7) | 33 (21.2) |  | 27 (21.3) | 10 (22.2) |  | 11 (50.0) | 18 (19.8) |  |
| Others | 0 (0.0) | 1 (0.6) |  | 0 (0.0) | 1 (2.2) |  | 0 (0.0) | 0 (0.0) |  |
| ***Size (cm)*** |  |  |  |  |  |  |  |  |  |
| 0.6 – 2.0 | 6 (14.6) | 12 (7.8) | *0.421* | 13 (10.5) | 4 (8.9) | *0.090* | 0 (0.0) | 9 (9.9) | *0.401* |
| 2.1 – 5.0 | 26 (63.4) | 94 (61.4) |  | 77 (62.1) | 23 (51.1) |  | 12 (57.1) | 52 (57.1) |  |
| 5.1 – 10.0 | 9 (22.0) | 45 (29.4) |  | 34 (27.4) | 16 (35.6) |  | 9 (42.9) | 28 (30.8) |  |
| > 10.0 | 0 (0.0) | 2 (1.3) |  | 0 (0.0) | 2 (4.4) |  | 0 (0.0) | 2 (2.2) |  |
| ***Macroscopic aspects*** |  |  |  |  |  |  |  |  |  |
| Ulcerative | 16 (37.2) | 45 (29.0) | *0.163* | 40 (31.5) | 10 (22.2) | *0.218* | 6 (27.3) | 28 (30.8) | *0.396* |
| Vegetative | 7 (16.3) | 39 (25.2) |  | 32 (25.2) | 12 (26.7) |  | 6 (27.3) | 28 (30.8) |  |
| Verrucous | 0 (0.0) | 11 (7.1) |  | 7 (5.5) | 2 (4.4) |  | 0 (0.0) | 7 (7.7) |  |
| Ulcer-vegetative | 11 (25.6) | 40 (25.8) |  | 27 (21.3) | 17 (37.8) |  | 6 (27.3) | 22 (24.2) |  |
| Others | 9 (20.9) | 20 (12.9) |  | 21 (16.5) | 4 (8.9) |  | 4 (18.2) | 6 (6.6) |  |
| ***Histological subtype*** |  |  |  |  |  |  |  |  |  |
| Usual | 29 (67.4) | 50 (31.8) | *< 0.0001* | 53 (41.4) | 12 (26.7) | *<0.0001* | 7 (37.8) | 37 (40.7) | *0.435* |
| Warty | 4 (9.3) | 54 (34.4) |  | 43 (33.6) | 9 (20.0) |  | 5 (22.7) | 27 (29.7) |  |
| Basaloid | 2 (4.7) | 6 (3.8) |  | 0 (0.0) | 8 (17.8) |  | 2 (9.1) | 4 (4.4) |  |
| Warty-basaloid | 2 (4.7) | 15 (9.6) |  | 5 (3.9) | 9 (20.0) |  | 1 (4.5) | 7 (7.7) |  |
| Mixed | 3 (7.0) | 29 (18.5) |  | 23 (18.0) | 7 (15.6) |  | 6 (27.3) | 15 (16.5) |  |
| Others | 3 (7.0) | 3 (1.9) |  | 4 (3.1) | 0 (0.0) |  | 1 (4.5) | 1 (1.1) |  |
| ***Histological subtype according HPV*** |  |  |  |  |  |  |  |  |  |
| HPV-no-associated | 32 (74.4) | 53 (33.8) | *< 0.0001* | 57 (44.5) | 12 (26.7) | *0.053* | 8 (36.4) | 38 (41.8) | *0.522* |
| HPV-associated | 8 (18.6) | 75 (47.8) |  | 48 (37.5) | 26 (57.8) |  | 8 (36.4) | 38 (41.8) |  |
| Mixed | 3 (7.0) | 29 (18.5) |  | 23 (18.0) | 7 (15.6) |  | 6 (27.3) | 15 (16.5) |  |
| ***Grade (G)*** |  |  |  |  |  |  |  |  |  |
| G1 | 6 (14.0) | 19 (12.1) | *0.123* | 19 (14.8) | 1 (2.2) | *0.008* | 1 (4.5) | 13 (14.3) | *0.555* |
| G2 | 11 (25.6) | 66 (42.0) |  | 52 (40.6) | 13 (28.9) |  | 8 (36.4) | 28 (30.8) |  |
| G3 | 26 (60.5) | 72 (45.9) |  | 57 (44.5) | 31 (68.9) |  | 13 (59.1) | 50 (54.9) |  |
| ***Angiolymphatic invasion*** |  |  |  |  |  |  |  |  |  |
| Absent | 29 (67.4) | 100 (63.7) | *0.721* | 87 (68.0) | 22 (48.9) | *0.031* | 9 (40.9) | 57 (62.6) | *0.091* |
| Present | 14 (32.6) | 57 (36.3) |  | 41 (32.0) | 23 (51.1) |  | 13 (59.1) | 34 (37.4) |  |
| ***Perineural invasion*** |  |  |  |  |  |  |  |  |  |
| Absent | 26 (60.5) | 101 (64.3) | *0.721* | 83 (64.8) | 26 (57.8) | *0.473* | 12 (54.5) | 56 (61.5) | *0.630* |
| Present | 17 (39.5) | 56 (35.7) |  | 45 (35.2) | 19 (42.2) |  | 10 (45.5) | 35 (38.5) |  |
| ***Tumor focus*** |  |  |  |  |  |  |  |  |  |
| Unifocal | 37 (88.1) | 141 (90.4) | *0.773* | 113 (89.0) | 39 (86.7) | *0.787* | 19 (86.4) | 78 (85.7) | *1.000* |
| Multifocal | 5 (11.9) | 15 (9.6) |  | 14 (11.0) | 6 (13.3) |  | 3 (13.6) | 13 (14.3) |  |
| ***Carcinoma in situ associated*** |  |  |  |  |  |  |  |  |  |
| Absent | 8 (18.6) | 28 (17.8) | *1.000* | 21 (16.4) | 9 (20.0) | *0.648* | 8 (36.4) | 13 (14.3) | *0.029* |
| Present | 35 (81.4) | 129 (82.2) |  | 107 (83.6) | 36 (80.0) |  | 14 (63.6) | 78 (85.7) |  |
| ***Sarcomatoid component*** |  |  |  |  |  |  |  |  |  |
| Absent | 36 (85.7) | 129 (82.2) | *0.818* | 106 (82.8) | 36 (80.0) | *0.657* | 20 (90.9) | 69 (75.8) | *0.153* |
| Present | 6 (14.3) | 28 (17.8) |  | 22 (17.2) | 9 (20.0) |  | 2 (9.1) | 22 (24.2) |  |
| ***Lichen sclerosus*** |  |  |  |  |  |  |  |  |  |
| Absent | 31 (75.6) | 110 (71.9) | *0.697* | 89 (71.2) | 36 (81.8) | *0.230* | 15 (71.4) | 68 (76.4) | *0.778* |
| Present | 10 (24.4) | 43 (28.1) |  | 36 (28.8) | 8 (18.2) |  | 6 (28.6) | 21 (23.6) |  |
| ***Primary tumor (T)*** |  |  |  |  |  |  |  |  |  |
| pT1 | 13 (30.2) | 32 (20.4) | *0.117* | 31 (24.2) | 7 (15.6) | *0.360* | 1 (4.5) | 16 (17.6) | *0.042* |
| pT2 | 14 (32.6) | 39 (24.8) |  | 35 (27.3) | 11 (24.4) |  | 3 (13.6) | 28 (30.8) |  |
| pT3 – pT4 | 16 (37.2) | 86 (54.8) |  | 62 (48.4) | 27 (60.0) |  | 18 (81.8) | 47 (51.6) |  |
| ***Stage*** |  |  |  |  |  |  |  |  |  |
| I | 12 (27.9) | 30 (19.1) | *0.470* | 29 (22.7) | 6 (13.3) | *0.232* | 1 (4.5) | 15 (16.5) | *0.072* |
| II | 28 (65.1) | 113 (72.0) |  | 90 (70.3) | 33 (73.3) |  | 16 (72.7) | 69 (75.8) |  |
| III - IV | 3 (7.0) | 14 (8.9) |  | 9 (7.0) | 6 (13.3) |  | 5 (22.7) | 7 (7.7) |  |
| ***Pattern of invasion*** |  |  |  |  |  |  |  |  |  |
| Expansive | 24 (66.7) | 116 (77.3) | *0.281* | 91 (75.2) | 33 (76.7) | *1.000* | 16 (76.2) | 64 (74.4) | *1.000* |
| Infiltrative | 13 (33.3) | 32 (21.3) |  | 28 (23.1) | 10 (23.3) |  | 5 (23.8) | 22 (25.6) |  |
| ***Tumor thickness (mm)*** |  | | | | | | | | |
| *<* 5.0 | 17 (45.9) | 56 (37.3) | *0.352* | 52 (42.6) | 16 (37.2) | *0.592* | 5 (23.8) | 34 (38.6) | *0.311* |
| > 5.0 | 20 (54.1) | 94 (62.7) |  | 70 (57.4) | 27 (62.8) |  | 16 (76.2) | 54 (61.4) |  |
| ***Depth of invasion (mm)*** |  | | | | | | | | |
| *<* 5.0 | 18 (48.6) | 79 (53.4) | *0.268* | 72 (52.9) | 17 (40.5) | *0.115* | 6 (28.6) | 47 (54.0) | *0.066* |
| 5.0 – 10.0 | 16 (43.2) | 45 (30.4) |  | 34 (24.9) | 17 (40.5) |  | 11 (52.4) | 24 (27.6) |  |
| > 10.0 | 3 (8.1) | 24 (16.2) |  | 16 (13.1) | 8 (19.0) |  | 4 (19.0) | 16 (18.4) |  |
| ***Lymph node metastasis*** |  | | | | | | | | |
| No | 2 (15.4) | 25 (41.0) | *0.115* | 17 (37.8) | 4 (23.5) | *0.375* | 4 (36.4) | 12 (34.3) | *1.000* |
| Yes | 11 (84.6) | 36 (59.0) |  | 28 (6.2) | 13 (76.5 |  | 7 (63.6) | 23 (65.7) |  |
| ***Extranodal extension*** |  | | | | | | | | |
| No | 3 (27.3) | 8 (21.6) | *0.697* | 7 (24.1) | 3 (23.1) | *1.000* | 0 (0.0) | 5 (20.8) | *0.562* |
| Yes | 8 (72.7) | 29 (78.4) |  | 22 (75.9) | 10 (76.9) |  | 7 (100.0) | 19 (79.2) |  |
| ***Ki-67 global expression*** |  | | | | | | | | |
| Low | 20 (60.6) | 107 (76.4) | *0.08* | 107 (83.6) | 20 (44.4) | *< 0.0001* | 16 (72.7) | 63 (72.4) | *1.000* |
| High | 13 (39.4) | 33 (23.6) |  | 21 (16.4) | 25 (55.6) |  | 6 (27.3) | 24 (27.6) |  |
| ***Ki-67 hotspot expression*** |  | | | | | | | | |
| Low | 10 (30.3) | 63 (45.0) | *0.170* | 67 (52.3) | 6 (13.3) | *< 0.0001* | 9 (40.9) | 35 (40.2) | *1.000* |
| High | 23 (69.7) | 77 (55.0) |  | 61 (47.7) | 39 (86.7) |  | 13 (59.1) | 52 (59.8) |  |
| ***P53 global expression*** |  | | | | | | | | |
| Negative | 11 (33.3) | 84 (60.0) | *0.007* | 68 (53.1) | 27 (60.0) | *0.488* | 13 (59.1) | 45 (51.7) | *0.635* |
| Positive | 22 (66.7) | 56 (40.0) |  | 60 (46.9) | 18 (40.0) |  | 9 (40.9) | 42 (48.3) |  |
| ***p53 stratified expression*** |  | | | | | | | | |
| Negative | 11 (33.3) | 84 (60.0) | *0.002* | 68 (53.1) | 27 (60.0) | *0.706* | 13 (59.1) | 45 (51.7) | *0.201* |
| Positive (+) | 8 (24.2) | 36 (25.7) |  | 33 (25.8) | 11 (24.4) |  | 3 (13.6) | 27 (31.0) |  |
| Positive (++) | 14 (42.4) | 20 (14.3) |  | 27 (21.1) | 7 (15.6) |  | 6 (27.3) | 15 (17.2) |  |
| ***P16 overexpression*** |  | | | | | | | | |
| No | 25 (75.8) | 103 (73.6) | *1.000* | - | - | *-* | 18 (81.8) | 62 (71.3) | *0.422* |
| Yes | 8 (24.2) | 37 (26.4) |  | - | - |  | 4 (18.2) | 25 (28.7) |  |
