# Supplementary material for: A comprehensive analysis of penile cancer in the region with the highest worldwide incidence reveals new insights into the disease
Source: BMC Cancer. 2022 Oct 15;22:1063. doi: 10.1186/s12885-022-10127-z (PMC9569053; doi:10.1186/s12885-022-10127-z)
Supplement: Supplementary file 3 — Additional file 3: Supplementary material (S3) Table 1. Statistical data for p53 and ki-67 protein expression analysis in penile cancer. [file 12885_2022_10127_MOESM3_ESM.docx]

**Supplementary material (S3)**

**Table 1**. Statistical data for p53 and ki-67 protein expression analysis in penile cancer

| **Parameters** | **P53** | | | ***p value*** | **Ki-67 global** | | ***p value*** | **Ki-67 hotspot** | | ***p value*** |
| --- | --- | --- | --- | --- | --- | --- | --- | --- | --- | --- |
|  | Negative | Positive (+) | Positive (++) |  | Negative | Positive |  | Low | High |  |
| ***Age*** | *N (%)* | *N (%)* | *N (%)* |  | *N (%)* | *N (%)* |  | *N (%)* | *N (%)* |  |
| 18 – 40 | 12 (12.8) | 7 (15.9) | 5 (14.7) | *0.614* | 16 (12.7) | 8 (17.4) | *0.231* | 7 (9.7) | 17 (7.0) | *0.222* |
| 41 – 60 | 24 (25.5) | 15 (34.1) | 12 (35.3) |  | 34 (27.0) | 17 (37.0) |  | 19 (26.4) | 32 (32.0) |  |
| > 60 | 58 (61.7) | 22 (50.0) | 17 (50.0) |  | 76 (60.3) | 21 (45.7) |  | 46 (63.9) | 51 (51.0) |  |
| ***Schooling*** |  | | | | | | | | |  |
| No education | 38 (79.2) | 19 (73.1) | 13 (81.2) | *0.963* | 48 (77.4) | 22 (78.6) | *0.108* | 28 (80.0) | 42 (76.4) | *0.713* |
| Primary school | 9 (18.8) | 6 (23.1) | 3 (18.8) |  | 14 (22.6) | 4 (14.3) |  | 7 (20.0) | 11 (20.0) |  |
| Secondary school | 1 (2.1) | 1 (3.8) | 0 (0.0) |  | 0 (0.0) | 2 (7.1) |  | 0 (0.0) | 2 (3.6) |  |
| ***Smoking habits*** |  | | | | | | | | |  |
| No | 25 (42.4) | 8 (25.8) | 13 (59.1) | *0.052* | 32 (39.5) | 14 (45.2) | *0.669* | 21 (46.7) | 25 (37.3) | *0.336* |
| Yes | 34 (57.6) | 23 (74.2) | 9 (40.9) |  | 49 (60.5) | 17 (54.8) |  | 24 (53.3) | 42 (62.7) |  |
| ***Alcoholism*** |  |  |  |  |  |  | *0.315* |  |  | *0.032* |
| No | 21 (48.8) | 7 (31.8) | 8 (66.7) | *0.148* | 28 (50.9) | 8 (36.4) |  | 18 (64.3) | 18 (36.7) |  |
| Yes | 22 (51.2) | 15 (68.2) | 4 (33.3) |  | 27 (49.1) | 14 (63.6) |  | 10 (35.7) | 31 (63.3) |  |
| ***Phimosis*** |  |  |  |  |  |  |  |  |  |  |
| No | 17 (31.5) | 6 (21.4) | 6 (30.0) | *0.647* | 19 (24.7) | 10 (40.0) | *0.201* | 10 (22.2) | 19 (33.3) | *0.271* |
| Yes | 37 (68.5) | 22 (78.6) | 14 (70.0) |  | 58 (75.3) | 15 (60.0) |  | 35 (77.8) | 38 (66.7) |  |
| ***Zoophilia*** |  |  |  |  |  |  |  |  |  |  |
| No | 14 (51.9) | 7 (38.9) | 5 (33.3) | *0.505* | 19 (44.2) | 7 (41.2) | *1.000* | 11 (45.8) | 15 (41.7) | *0.795* |
| Yes | 13 (48.1) | 11 (61.1) | 10 (66.7) |  | 24 (55.8) | 10 (58.8) |  | 13 (54.2) | 21 (58.3) |  |
| ***Genital hygiene*** |  |  |  |  |  |  |  |  |  |  |
| Good | 13 (36.1) | 3 (15.8) | 5 (27.8) | *0.292* | 13 (23.2) | 8 (47.1) | *0.072* | 9 (28.1) | 12 (29.3) | *1.000* |
| Poor or moderate | 23 (63.9) | 16 (84.2) | 13 (72.2) |  | 43 (76.8) | 9 (52.9) |  | 23 (71.9) | 29 (70.7) |  |
| ***STD history*** |  |  |  |  |  |  |  |  |  |  |
| No | 14 (43.8) | 9 (47.4) | 10 (66.7) | *0.351* | 23 (46.0) | 10 (62.5) | *0.389* | 14 (46.7) | 19 (52.8) | *0.805* |
| Yes | 18 (56.2) | 10 (52.6) | 5 (33.3) |  | 27 (54.0) | 6 (37.5) |  | 16 (53.3) | 17 (47.2) |  |
| ***N° sexual partners*** |  |  |  |  |  |  |  |  |  |  |
| < 6 | 9 (31.0) | 3 (20.0) | 5 (35.7) | *0.873* | 13 (29.5) | 4 (28.6) | *0.920* | 4 (16.7) | 13 (38.2) | *0.036* |
| 6 – 10 | 5 (17.2) | 4 (26.7) | 2 (14.3) |  | 9 (20.5) | 2 (14.3) |  | 8 (33.3) | 3 (8.8) |  |
| > 10 | 15 (51.7) | 8 (53.3) | 7 (50.0) |  | 22 (50.0) | 8 (57.1) |  | 12 (50.0) | 18 (52.9) |  |
| ***Symptoms onset*** |  |  |  | *0.662* |  |  | *0.068* |  |  | *1.000* |
| 0 – 12 months | 25 (55.6) | 18 (62.1) | 14 (66.7) |  | 36 (53.7) | 21 (75.0) |  | 24 (60.0) | 33 (60.0) |  |
| > 12 months | 20 (44.4) | 11 (37.9) | 7 (33.3) |  | 31 (46.3) | 7 (25.0) |  | 16 (40.0) | 22 (40.0) |  |
| ***Surgical procedure*** |  |  |  |  |  |  |  |  |  |  |
| Preservative (exeresis) | 3 (3.2) | 2 (4.5) | 0 (0.0) | *0.877* | 4 (3.1) | 1 (2.2) | *0.939* | 2 (2.7) | 3 (3.0) | *0.905* |
| Total glansectomy | 4 (4.2) | 2 (4.5) | 1 (2.9) |  | 6 (4.7) | 1 (2.2) |  | 3 (4.1) | 4 (4.0) |  |
| Partial penectomy | 70 (73.7) | 29 (65.9) | 27 (79.4) |  | 90 (70.9) | 36 (78.3) |  | 55 (75.3) | 71 (71.0) |  |
| Total penectomy | 11 (11.6) | 9 (20.5) | 4 (11.8) |  | 19 (15.0) | 5 (10.9) |  | 10 (13.7) | 14 (14.0) |  |
| Emasculation | 7 (7.4) | 2 (4.5) | 2 (5.9) |  | 8 (6.3) | 3 (6.5) |  | 3 (4.1) | 8 (8.0) |  |
| ***Topography*** |  |  |  |  |  |  |  |  |  |  |
| Foreskin and/or coronal sulcus | 4 (4.3) | 3 (6.8) | 0 (0.0) | *0.457* | 6 (4.7) | 1 (2.2) | *0.046* | 4 (5.5) | 3 (3.0) | *0.839* |
| Glans | 46 (48.9) | 18 (40.9) | 11 (32.4) |  | 61 (48.0) | 14 (31.1) |  | 34 (46.6) | 41 (41.4) |  |
| Corpus | 3 (3.2) | 0 (0.0) | 2 (5.9) |  | 2 (1.6) | 3 (6.7) |  | 2 (2.7) | 3 (3.0) |  |
| Glans + foreskin and/or coronal sulcus | 22 (23.4) | 14 (31.8) | 11 (32.4) |  | 30 (23.6) | 17 (37.8) |  | 17 (23.3) | 30 (30.3) |  |
| Glans + corpus | 18 (19.1) | 9 (20.5) | 10 (29.4) |  | 28 (22.0) | 9 (20.0) |  | 16 (21.9) | 21 (21.2) |  |
| Others | 1 (1.1) | 0 (0.0) | 0 (0.0) |  | 0 (0.0) | 1 (2.2) |  | 0 (0.0) | 1 (1.0) |  |
| ***Size (cm)*** |  |  |  |  |  |  |  |  |  |  |
| 0.6 – 2.0 | 8 (8.7) | 3 (6.8) | 6 (18.2) | *0.404* | 16 (12.9) | 1 (2.2) | *0.015* | 6 (8.5) | 11 (11.2) | *0.674* |
| 2.1 – 5.0 | 52 (56.6) | 27 (61.4) | 21 (63.6) |  | 69 (55.6) | 31 (68.9) |  | 42 (59.2) | 58 (59.2) |  |
| 5.1 – 10.0 | 31 (33.7) | 13 (29.5) | 6 (18.2) |  | 39 (31.5) | 11 (24.4) |  | 23 (32.4) | 27 (27.6) |  |
| > 10.0 | 1 (1.1) | 1 (2.3) | 0 (0.0) |  | 0 (0.0) | 2 (4.4) |  | 0 (0.0) | 2 (2.0) |  |
| ***Macroscopic aspects*** |  |  |  |  |  |  |  |  |  |  |
| Ulcerative | 26 (27.7) | 13 (29.5) | 11 (32.4) | *0.505* | 40 (31.7) | 10 (21.7) | *0.199* | 18 (24.7) | 32 (32.3) | *0.028* |
| Vegetative | 24 (25.5) | 14 (31.8) | 6 (17.6) |  | 33 (26.2) | 11 (23.9) |  | 24 (32.9) | 20 (20.2) |  |
| Verrucous | 6 (6.4) | 3 (6.8) | 0 (0.0) |  | 7 (5.6) | 2 (4.3) |  | 6 (8.2) | 3 (3.0) |  |
| Ulcer-vegetative | 22 (23.4) | 11 (25.0) | 11 (32.4) |  | 26 (20.6) | 18 (39.1) |  | 12 (16.4) | 32 (32.3) |  |
| Others | 16 (17.0) | 3 (6.8) | 6 (17.6) |  | 20 (15.9) | 5 (10.9 |  | 13 (17.8) | 12 (12.1) |  |
| ***Histological subtype*** |  |  |  |  |  |  |  |  |  |  |
| Usual | 28 (29.5) | 14 (31.8) | 23 (67.6) | *0.001* | 43 (33.9) | 22 (47.8) | *< 0.0001* | 22 (30.1) | 43 (43.0) | *< 0.0001* |
| Warty | 35 (36.8) | 15 (34.1) | 2 (5.9) |  | 50 (39.4) | 2 (4.3) |  | 38 (52.1) | 14 (14.0) |  |
| Basaloid | 6 (6.3) | 2 (4.5) | 0 (0.0) |  | 0 (0.0) | 8 (17.4) |  | 0 (0.0) | 8 (8.0) |  |
| Warty-basaloid | 10 (10.5) | 3 (6.8) | 1 (2.9) |  | 7 (5.5) | 7 (15.2) |  | 0 (0.0) | 14 (14.0) |  |
| Mixed | 12 (12.6) | 10 (22.7) | 8 (23.5) |  | 23 (18.1) | 7 (15.2) |  | 11 (15.1) | 19 (19.0) |  |
| Others | 4 (4.2) | 0 (0.0) | 0 (0.0) |  | 4 (3.1) | 0 (0.0) |  | 2 (2.7) | 2 (2.0) |  |
| ***Histological subtype according HPV*** |  |  |  |  |  |  |  |  |  |  |
| HPV-no-associated | 32 (33.7) | 14 (31.8) | 23 (67.6) | *< 0.0001* | 47 (37.0) | 22 (47.8) | *0.477* | 24 (32.9) | 45 (45.0) | *0.116* |
| HPV-associated | 51 (53.7) | 20 (45.5) | 3 (8.8) |  | 57 (44.9) | 17 (37.0) |  | 38 (52.1) | 36 (36.0) |  |
| Mixed | 12 (12.6) | 10 (22.7) | 8 (23.5) |  | 23 (18.1) | 7 (15.2) |  | 11 (15.1) | 19 (19.0) |  |
| ***Grade (G)*** |  |  |  |  |  |  |  |  |  |  |
| G1 | 17 (17.9) | 3 (6.8) | 0 (0.0) | *< 0.0001* | 19 (15.0) | 1 (2.2) | *<0.0001* | 14 (19.2) | 6 (6.0) | *< 0.0001* |
| G2 | 38 (40.0) | 21 (47.7) | 6 (17.6) |  | 59 (46.5) | 6 (13.0) |  | 43 (58.9) | 22 (22.0) |  |
| G3 | 40 (42.1) | 20 (45.5) | 28 (82.4) |  | 49 (38.6) | 39 (84.8) |  | 16 (21.9) | 72 (72.0) |  |
| ***Angiolymphatic invasion*** |  |  |  |  |  |  |  |  |  |  |
| Absent | 65 (68.4) | 28 (63.6) | 16 (47.1) | *0.093* | 9 (70.9) | 19 (41.3) | *0.001* | 54 (74.0) | 55 (55.0) | *0.011* |
| Present | 30 (31.6) | 16 (36.4) | 18 (52.9) |  | 37 (29.1) | 27 (58.7) |  | 19 (26.0) | 45 (45.0) |  |
| ***Perineural invasion*** |  |  |  |  |  |  |  |  |  |  |
| Absent | 66 (69.5) | 28 (63.6) | 15 (44.1) | *0.036* | 86 (67.7) | 23 (50.0) | *0.049* | 57 (78.1) | 52 (52.0) | *< 0.0001* |
| Present | 29 (30.5) | 16 (36.4) | 19 (55.9) |  | 41 (32.3) | 23 (50.0) |  | 16 (21.9) | 48 (48.0) |  |
| ***Tumor focus*** |  |  |  |  |  |  |  |  |  |  |
| Unifocal | 84 (89.4) | 40 (90.9) | 28 (82.4) | *0.450* | 114 (90.5) | 38 (82.6) | *0.181* | 66 (91.7) | 86 (86.0) | *0.337* |
| Multifocal | 10 (10.6) | 4 (90.1) | 6 (17.6) |  | 12 (9.5) | 8 (17.4) |  | 6 (8.3) | 14 (14.0) |  |
| ***Carcinoma in situ associated*** |  |  |  |  |  |  |  |  |  |  |
| Absent | 14 (14.7) | 5 (11.4) | 11 (32.4) | *0.041* | 20 (15.7) | 10 (21.7) | *0.369* | 11 (15.1) | 19 (19.0) | *0.547* |
| Present | 81 (85.3) | 39 (88.6) | 23 (67.6) |  | 107 (84.3) | 36 (78.3) |  | 62 (84.9) | 81 (81.0) |  |
| ***Sarcomatoid component*** |  |  |  |  |  |  |  |  |  |  |
| Absent | 80 (84.2) | 36 (81.8) | 26 (76.5) | *0.555* | 111 (87.4) | 31 (77.4) | *0.006* | 68 (93.2) | 74 (74.0) | *0.001* |
| Present | 15 (15.8) | 8 (18.2) | 8 (23.5) |  | 16 (12.6) | 15 (32.6) |  | 5 (6.8) | 26 (26.0) |  |
| ***Lichen sclerosus*** |  |  |  |  |  |  |  |  |  |  |
| Absent | 70 (76.1) | 27 (62.8) | 28 (82.4) | *0.132* | 90 (72.6) | 35 (77.8) | *0.557* | 48 (65.8) | 77 (80.2) | *0.051* |
| Present | 22 (23.9) | 16 (37.2) | 6 (17.6) |  | 34 (27.4) | 10 (22.2) |  | 25 (34.2) | 19 (19.8) |  |
| ***Primary tumor (T)*** |  |  |  | *0.031* |  |  | *0.031* |  |  | *0.014* |
| pT1 | 29 (30.5) | 7 (15.9) | 2 (5.9) |  | 34 (26.8) | 4 (8.7) |  | 24 (32.9) | 14 (14.0) |  |
| pT2 | 22 (23.2) | 13 (29.5) | 11 (32.4) |  | 32 (25.2) | 14 (30.4) |  | 17 (23.3) | 29 (29.0) |  |
| pT3 – pT4 | 44 (46.3) | 24 (54.5) | 21 (61.8) |  | 61 (48.0) | 28 (60.9) |  | 32 (43.8) | 57 (57.0) |  |
| ***Stage*** |  |  |  |  |  |  |  |  |  |  |
| I | 27 (28.4) | 6 (13.6) | 2 (5.9) | *0.020* | 31 (24.4) | 4 (8.7) | *0.038* | 22 (30.1) | 13 (13.0) | *0.002* |
| II | 59 (62.1) | 36 (81.8) | 28 (82.4) |  | 87 (68.5) | 36 (78.3) |  | 49 (67.1) | 74 (74.0) |  |
| III - IV | 9 (9.5) | 2 (4.5) | 4 (11.8) |  | 9 (7.1) | 6 (13.0) |  | 2 (2.7) | 13 (13.0) |  |
| ***Koilocytosis*** |  |  |  |  |  |  |  |  |  |  |
| Absent | 11 (11.6) | 8 (18.2) | 14 (41.2) | *0.002* | 20 (15.7) | 13 (28.3) | *0.080* | 10 (13.7) | 23 (23.0) | *0.170* |
| Present | 84 (88.4) | 36 (81.8) | 20 (58.8) |  | 107 (84.3) | 33 (71.7) |  | 63 (86.3) | 77 (77.0) |  |
| ***PCR HPV*** |  |  |  |  |  |  |  |  |  |  |
| Negative | 13 (22.4) | 3 (10.0) | 6 (28.6) | *0.202* | 16 (20.3) | 6 (20.0) | *1.000* | 9 (20.5) | 13 (20.0) | *1.000* |
| Positive | 45 (77.6) | 27 (90.0) | 15 (71.4) |  | 63 (79.7) | 24 (80.0) |  | 35 (79.5) | 52 (80.0) |  |
| ***Pattern of invasion*** |  |  |  |  |  |  |  |  |  |  |
| Expansive | 74 (82.2) | 34 (81.0) | 16 (50.0) | *0.002* | 101 (82.1) | 23 (56.1) | *0.001* | 61 (87.1) | 63 (67.0) | *<0.0001* |
| Infiltrative | 14 (15.6) | 8 (19.0) | 16 (50.0) |  | 20 (16.3) | 18 (43.8) |  | 7 (10.0) | 31 (33.0) |  |
| Others | 2 (2.2) | 0 (0.0) | 0 (0.0) |  | 2 (1.6) | 0 (0.0) |  | 2 (2.9) | 0 (0.0) |  |
| ***Tumor thickness (mm)*** |  | | | | | | | | |  |
| *<* 5.0 | 40 (44.9) | 17 (39.5) | 11 (33.3) | *0.511* | 54 (43.9) | 14 (33.3) | *0.277* | 34 (48.6) | 34 (35.8) | *0.111* |
| > 5.0 | 49 (55.1) | 26 (60.5) | 22 (66.7) |  | 69 (56.1) | 28 (66.7) |  | 36 (51.4) | 61 (64.2) |  |
| ***Depth of invasion (mm)*** |  | | | | | | | | |  |
| *<* 5.0 | 56 (62.9) | 21 (50.0) | 12 (36.4) | *0.072* | 73 (59.8) | 16 (38.1) | *0.034* | 45 (64.3) | 44 (46.8) | *0.084* |
| 5.0 – 10.0 | 22 (24.7) | 13 (31.0) | 16 (48.5) |  | 32 (26.2) | 19 (45.2) |  | 18 (25.7) | 33 (35.1) |  |
| > 10.0 | 11 (12.4) | 8 (19.0) | 5 (15.2) |  | 11 (13.9) | 7 (16.7) |  | 7 (10.0) | 17 (18.1) |  |
| ***Lymph node metastasis*** |  | | | | | | | | |  |
| No | 14 (43.8) | 6 (37.5) | 1 (7.1) | *0.046* | 19 (46.3) | 2 (9.5) | *0.004* | 16 (72.7) | 5 (12.5) | *< 0.0001* |
| Yes | 18 (56.2) | 10 (62.5) | 13 (92.9) |  | 22 (53.7) | 19 (90.5) |  | 6 (27.3) | 35 (87.5) |  |
| ***Extranodal extension*** |  | | | | | | | | |  |
| No | 7 (36.8) | 2 (20.0) | 1 (7.7) | *0.190* | 8 (34.8) | 2 (10.5) | *0.083* | 3 (42.9) | 7 (20.0) | *0.328* |
| Yes | 12 (63.2) | 8 (80.0) | 12 (92.3) |  | 15 (65.2) | 17 (89.5) |  | 4 (57.1) | 28 (80.0) |  |
| ***Ki-67 global expression*** |  | | | | | | | | |  |
| Low | 75 (78.9) | 34 (77.3) | 18 (52.9) | *0.015* | - | - | *-* | - | - | *-* |
| High | 20 (21.1) | 10 (22.7) | 16 (47.1) |  | - | - |  | - | - |  |
| ***Ki-67 hotspot expression*** |  | | | | | | | | |  |
| Low | 48 (50.5) | 19 (43.2) | 6 (17.6) | *0.004* | - | - | *-* | - | - | *-* |
| High | 47 (49.5) | 25 (56.8) | 28 (82.4) |  | - | - |  | - | - |  |
| ***P53 stratified expression*** |  | | | | | | | | |  |
| Negative | - | - | - | *-* | 75 (59.1) | 20 (43.5) | *0.013* | 48 (65.8) | 47 (47.0) | *0.002* |
| Positive (+) | - | - | -- |  | 34 (26.8) | 10 (21.7) |  | 19 (26.0) | 25 (25.0) |  |
| Positive (++) | - | - | - |  | 18 (14.2) | 16 (34.8) |  | 6 (8.2) | 28 (28.0) |  |
| ***P16 overexpression*** |  | | | | | | | | |  |
| No | 68 (71.6) | 33 (75.0) | 27 (79.4) | *0.715* | 107 (84.3) | 21 (45.7) | *< 0.0001* | 67 (91.8) | 61 (61.0) | *< 0.0001* |
| Yes | 27 (28.4) | 11 (25.0) | 7 (20.6) |  | 20 (15.7) | 25 (54.3) |  | 6 (8.2) | 39 (39.0) |  |
